# Hand-worn devices for assessment and rehabilitation of motor function and their potential use in BCI protocols: a review

**DOI:** 10.3389/fnhum.2023.1121481

**Published:** 2023-07-06

**Authors:** Madison Bates, Sridhar Sunderam

**Affiliations:** Neural Systems Lab, F. Joseph Halcomb III, M.D. Department of Biomedical Engineering, University of Kentucky, Lexington, KY, United States

**Keywords:** hand function impairments, functional assessment, brain-computer interface, assistive devices, sensor gloves, monitoring devices, neurorehabilitation

## Abstract

**Introduction:**

Various neurological conditions can impair hand function. Affected individuals cannot fully participate in activities of daily living due to the lack of fine motor control. Neurorehabilitation emphasizes repetitive movement and subjective clinical assessments that require clinical experience to administer.

**Methods:**

Here, we perform a review of literature focused on the use of hand-worn devices for rehabilitation and assessment of hand function. We paid particular attention to protocols that involve brain-computer interfaces (BCIs) since BCIs are gaining ground as a means for detecting volitional signals as the basis for interactive motor training protocols to augment recovery. All devices reviewed either monitor, assist, stimulate, or support hand and finger movement.

**Results:**

A majority of studies reviewed here test or validate devices through clinical trials, especially for stroke. Even though sensor gloves are the most commonly employed type of device in this domain, they have certain limitations. Many such gloves use bend or inertial sensors to monitor the movement of individual digits, but few monitor both movement and applied pressure. The use of such devices in BCI protocols is also uncommon.

**Discussion:**

We conclude that hand-worn devices that monitor both flexion and grip will benefit both clinical diagnostic assessment of function during treatment and closed-loop BCI protocols aimed at rehabilitation.

## 1. Introduction

Many neuromuscular and neurovascular disorders can affect a person's hand function, which in turn hinders activities of daily living (ADLs). The most common causes include stroke and spinal cord injury (SCI) (National Center for Chronic Disease Prevention and Health Promotion and Prevention and DFHDAS, 2023). More than 50% of stroke survivors over the age of 60 have reduced mobility and within that group, 85% have upper extremity (UE) impairments (Parker et al., 1986; Benjamin et al., 2017). For SCI, 59% of cases lead to tetraplegia, which can impair UE function (Center NSCIS, 2017). Impaired individuals must undergo extensive rehabilitation to attempt to regain hand function. Clinical functional assessment and treatment planning is critical for these individuals, but extremely difficult since the human hand and wrist having 21 degrees of freedom (DOF) controlled by 29 muscles and various cortical and subcortical structures (Jones and Lederman, 2006). The key brain structures for planning and executing volitional movement are the primary and pre-motor cortex, which have direct connections to spinal motoneurons (Nowak, 2008; Balasubramanian et al., 2010). Due to the complexity of these structures and neural connections, clinicians first assess the nature and level of impairment and then prescribe the rehabilitation strategy most likely to improve hand function.

In common clinical practice, the severity of impairment is assessed using functional tests, of which there is a wide variety (e.g., Fugl-Meyer Assessment, Nine Hole Peg Test of Finger Dexterity, etc.) (Fugl-Meyer et al., 1975; Mathiowetz et al., 1985). However, these functional assessments employ simplistic measures (e.g., time to complete a motor task or an integer rating from 0 to 2) that require clinical experience to apply and are time consuming (Ding et al., 2022). Some current developmental efforts focus on creating devices to monitor and assess hand function without the need for clinical expertise. Such sensor-based devices could provide objective, quantitative, time-efficient, and low-cost alternatives to the status quo. This review covers a variety of sensor-based devices that have the potential to revolutionize the assessment of hand function.

After assessing the severity of impairment, the clinician can then select an appropriate rehabilitation strategy. Extensive and timely physical therapy is usually indicated, which requires clinical expertise to administer (Good and Sawaki, 2010). Current therapies focus on harnessing neuroplasticity, drawing from research showing that patients can bolster their existing motoneuron connections and rewire their brains to develop new connections (Kleim and Jones, 2008; Janarthanan et al., 2020). Neuroplasticity-targeted rehabilitation uses repetitive functional tasks to stimulate the brain and reinforce neural pathways between affected motor units and the central nervous system (Goh et al., 2020). One way to initiate these repetitive movements is through functional electrical stimulation (FES) of hand muscles. FES devices are typically placed on the forearm muscles to induce hand flexion or extension. In our review, cases of FES being applied directly to the hand were also found. This approach might provide better control of fine hand movements.

**Figure 1:**
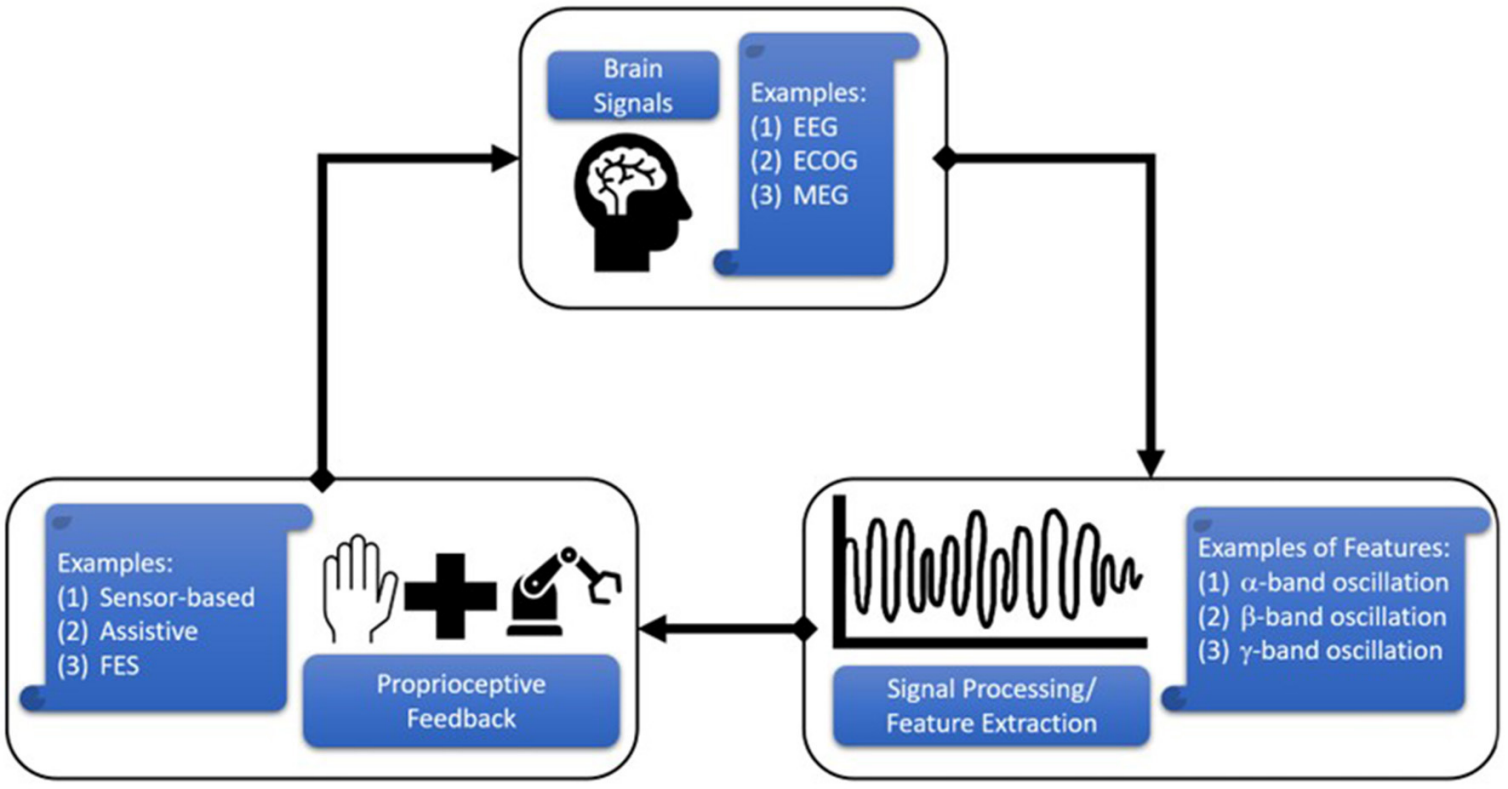
Schematic of Brain Computer Interface (BCI) operation in the context of this review. Brain-generated signals are acquired through electroencephalography (EEG), electrocorticography (ECOG), magnetoencephalography (MEG), etc., and processed to extract key features related to hand movement (e.g., alpha modulation). These features can be used to trigger or synced a hand-worn device (e.g., assistive exoskeleton or sensor glove) to provide proprioceptive feedback to the subject.

Brain-computer interfaces (BCIs) can also be used to promote neuroplasticity. Many BCIs in research are unidirectional, which means that they either send information from the brain via sensors to an external device (e.g., assistive devices or FES devices) (Crea et al., 2018; Cheng et al., 2020; Schwarz et al., 2020) or from the external device (e.g., sensor-based devices such as a Linderman et al.'s EMG glove) (Linderman and Rupasov, 2012; Bouton et al., 2016) to the brain through direct brain stimulation (Lobel and Lee, 2014; Niemeyer, 2016; Semprini et al., 2016). Research over the past decade has focused on the latter approach to provide proprioceptive feedback. Figure 1 depicts the close-loop system that is created by connecting brain signals with proprioceptive feedback. Signal processing techniques are used to extract features related to sensorimotor function (e.g., mu-beta EEG power), which are then used to trigger or sync with the proprioceptive device. These proprioceptive devices may either assist hand movement (assistive device), stimulate muscles (FES devices), or monitor and display sensor measurements. Assistive devices—either end-effector robots or exoskeletal/hand-worn devices—are the ones most commonly integrated with BCIs. End-effector robots are typically not portable, since they are fixed to a table or the ground. Therefore, the person using this device cannot move around. The end-effectors alleviate muscle fatigue by providing support while the patient focuses on physical movements of the hand (Balasubramanian et al., 2010). On the other hand, exoskeletal devices allow the patient to move around, but are rigidly attached to the hand and use kinematic models or a controller to guide the hand and fingers. This review found many different assistive devices in these two broad categories, but only a few of which—either assistive or sensor-based—were used in conjunction with BCIs.

The initial goal of this review was to determine the gaps in research or development related to hand-worn devices meant for rehabilitation or assessment of hand function in impaired individuals. With the belief that BCI systems offer a new and expanding frontier for rehabilitation, we further investigated avenues for research and development on the integration of different types of hand-worn devices (e.g., sensor-based, assistive) with brain activity monitoring (BAM).

## 2. Methods

### 2.1. Data sources and search strategy

We followed the guidelines of a systematic review, but we critiqued the devices in question rather than the studies in which they were used. We used the Preferred Reporting Items for Systematic Reviews and Meta-Analyses (PRISMA) as a frame of reference for the initial searches and then applied further exclusion criteria. The databases searched were PubMed, Medline, Espacenet, and Google Patents: PubMed and Medline for peer-reviewed articles, and Espacenet and Google Patents for device patents specifically related to hand impairments. All references published from 2002 to 2022 and originally written in English were considered. For each database, four individual searches were run using key terms found in Tables 1, 2. These tables show how the addition of each term narrows the pool of articles and patents. All the terms were separated with a comma, which served as a conjunction: the results are therefore references that include all the key terms; changing the order of the terms does not change the outcome. Some terms were repeated within the four searches on each database. The difference between the searches is the additive terms applied (i.e., “sensor” or “impaired hand function”). These additional terms allowed new references to be included in the analysis.

**Table 1:** Peer-reviewed article databases and search terms for the general systematic review of hand-worn devices.

**Peer-reviewed articles (July 22, 2022)**			
**PubMed**		**Medline**	
**1st search**		**1st search**	
Functional assessment	1,816,494	Neurorehabilitation	13,160
+ Motor function	73,820	+ Glove	29
+ Measurements	30,671		
+ Hand	3,394		
+ Glove	53		
+ Neurorehabilitation	21		
**2nd search**		**2nd search**	
Functional assessment	1,816,494	Neurorehabilitation	13,160
+ Motor function	73,820	+ Hand impairments	37
+ Sensor glove	24		
**3rd search**		**3rd search**	
Functional assessment	1,816,494	Motor function assessment	1,158
+ Motor function	73,820	+ Stroke	428
+ Impaired hand function	1,692	+ Sensors	27
+ Rehabilitation	830		
+ Sensors	29		
**4th search**		**4th search**	
Impaired motor function	47,204	Function assessment	25,344
+ Neurorehabilitation	3,303	+ Rehabilitation	2,795
+ Glove	32	+ Glove	16

Each additional search term narrows the number of references found within the database.

**Table 2:** Patent databases and search terms for the general systematic review of hand-worn devices.

**Patents (July 29, 2022)**			
**Espacenet**		**Google patents**	
**1st search**		**1st search**	
Functional assessment	269,358	Functional assessment	135,828
+ Hand	119,624	+ Hand	135,828
+ Sensors	26,089	+ Sensors	135,828
+ Gloves	998	+ Gloves	39,984
+ Neurorehabilitation	6	+ Neurorehabilitation	29
**2nd search**		**2nd search**	
Functional assessment Tools	62,446	Functional assessment Tools	135,828
+ Hand motor function	7,945	+ Hand motor function	135,828
+ Stroke	3,579	+ Stroke	107,495
+ Sensor glove	212	+ Sensor glove	4,549
+ Tracking recovery progress	71	+ Tracking recovery progress	1,170
		+ Rehabilitation	246
		+ BCI	23
**3rd search**		**3rd search**	
Impaired hand function	398,888	Impaired hand function	135,828
+ Stroke rehabilitation	2,391	+ Stroke rehabilitation	82,936
+ Gloves	176	+ Gloves	3,389
+ Functional assessment	55	+ Functional assessment	1,934
		+ Motor function	1,090
		+ Assistive device	207
		+ Finer finger movement	61
**4th search**		**4th search**	
Neurorehabilitation	1,204	Neurorehabilitation	4,095
+ Sensor glove	25	+ Sensor glove	52

Each additional search term narrows the number of references found within the database.

We performed an additional review to narrow the scope to hand-worn devices that were used or designed to be used in conjunction with a BCI. This also followed the PRISMA framework and then applied further exclusion criteria but only on PubMed. All references from 2009 to 2023 and originally written in English were considered. Four individual searches were run using the key terms in Table 3, which shows how the addition of each term narrows the pool. All the terms were separated with a comma, which served as a conjunction: the results are therefore references that include all the key terms; changing the order of these terms does not change the outcome. The first and second searches only focused on clinical trial articles, while the third and fourth search expanded the scope by including randomized controlled trials, review, and systematic reviews.

**Table 3:** Search terms for the secondary systematic review, which focuses on hand-worn devices used with BCIs.

**PubMed (January 16th, 2023)**			
**Type of articles: clinical trial**		**Type of articles: clinical trial, randomized controlled trial, review, systematic review**	
**Search 1**		**Search 1**	
Brain computer interface	83	Brain computer interface	418
+ Functional assessment	20	+ Functional assessment	50
+ Rehabilitation	10	+ Rehabilitation	29
**Search 2**		**Search 2**	
Brain computer interface	83	Brain computer interface	418
+ Hand	22	+ Hand	40
		+ Device	27

Each additional search term narrows the number of references found within the database.

### 2.2. Inclusion criteria

To be included in this review, references had to be peer-reviewed articles and/or approved patents focusing specifically on devices designed to aid in rehabilitation and functional assessment for individuals with impaired hand function. Any references that were not open-access were obtained through an interlibrary loan service (ILLiad) from the University of Kentucky (UK) Libraries.

### 2.3. Exclusion criteria

After the initial searches, additional criteria were applied to narrow down the relevant devices. References were excluded if they were outside the topic of interest (e.g., dental, neonatal, lockdown syndrome patients) or about devices that could not be worn on the hand. For example, functional electrical stimulation of the forearm muscles (and not those on the hand), smart watches, and wristbands were excluded since they do not directly interact with the hand or fingers. Articles focusing more on the rehabilitation protocol than on utilization of the hand-worn device were also excluded. Additionally, references not accessible within our means could not be included. Finally, if there were multiple articles on the same device, the most recent one was included and the earlier ones excluded if they did not add useful information, such as the accuracy of the sensors or technology in question. The most recent articles provided the best representation of the devices and their capabilities.

### 2.4. Reference selection and data extraction

The references in Tables 1–3 were processed through specific criteria stated in Sections 2.1, 2.3. The abstract and discussion sections of each reference were read, and all duplicates removed. The remaining references from the original review (*n* = 81) were read through completely and categorized. Data extraction focused on the reference source, year and type; the device type; device testing; and type of participants. References were sorted into Research Articles, Literature Reviews, Conference Papers, and Patents. All literature reviews were not included in the Data Analysis Tables since they mentions a large number of devices—some of which were duplicates from the Research Articles and Conference Papers. Therefore, the total number of references analyzed in this general review was 78. All devices belonged to the following seven categories: sensor-based, assistive, FES-based, passive (i.e., devices that give mechanical support only), FES-cum-sensor-based, sensor-cum-assistive, and FES-cum-assistive. The device testing section sorts all the references into clinical and non-clinical piles. The clinical category focuses on participants grouped by health status, and when applicable, the number of participants of each type.

Additionally, references remaining in the secondary review (*n* = 13) followed a similar selection process and were categorized by: Research Article, Literature Review, and Conference Paper. Since 12 out of the 13 were Literature Reviews. The authors took a different approach to the data analysis. They used devices in the Literature Reviews as the focus rather than the review themselves. The same exclusion and inclusion criteria (refer to Sections 2.2, 2.3) were applied to each research article mentioned in these reviews. The remaining references (*n* = 32) were analyzed and categorized as either BCI-assistive, BCI-FES, BCI-sensor-based, or a combination of those.

### 2.5. Data analysis

Devices were evaluated on the basis of their components and capabilities. Supplementary Table IV identifies and describes key characteristics (i.e., instrumentation, targeted body part, etc.) of the devices from the primary review. From the secondary review, Supplementary Table V breaks down these systems into the type of hand-worn device (instrumentation included), the BAM device, the purpose of the study, their results, and the limitations of the hand-worn device. The goal is to identify missing component configurations and instrumentation that have not yet been thoroughly researched or fully developed. These missing tools have the potential to further augment rehabilitation or functional assessment of hand impairments.

## 3. Results

### 3.1. General review of hand-worn devices

#### 3.1.1. Study selection, characteristics, and populations

**Figure 2:**
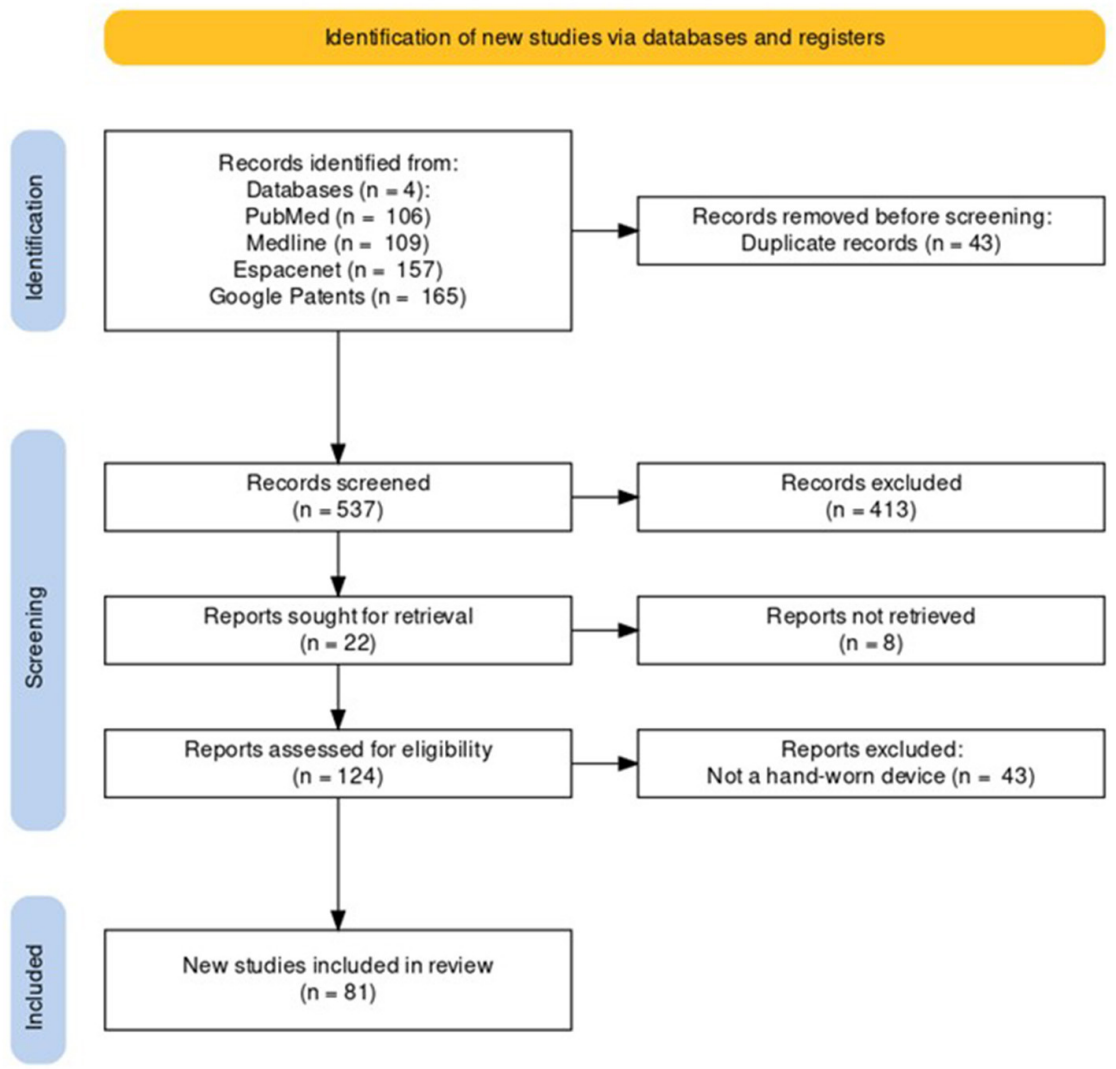
PRISMA flowchart for the Hand-Worn Device Review for rehabilitation and functional assessment for individuals suffering from impaired hand function. This review conducted searches in four different databases to search for literature (i.e., research articles, patents, other reviews) that focus on hand-worn devices. From the original 537 references found, only 81 of those references remained after exclusion criteria (i.e., must be a device attached to the hand) were applied.

**Figure 3:**
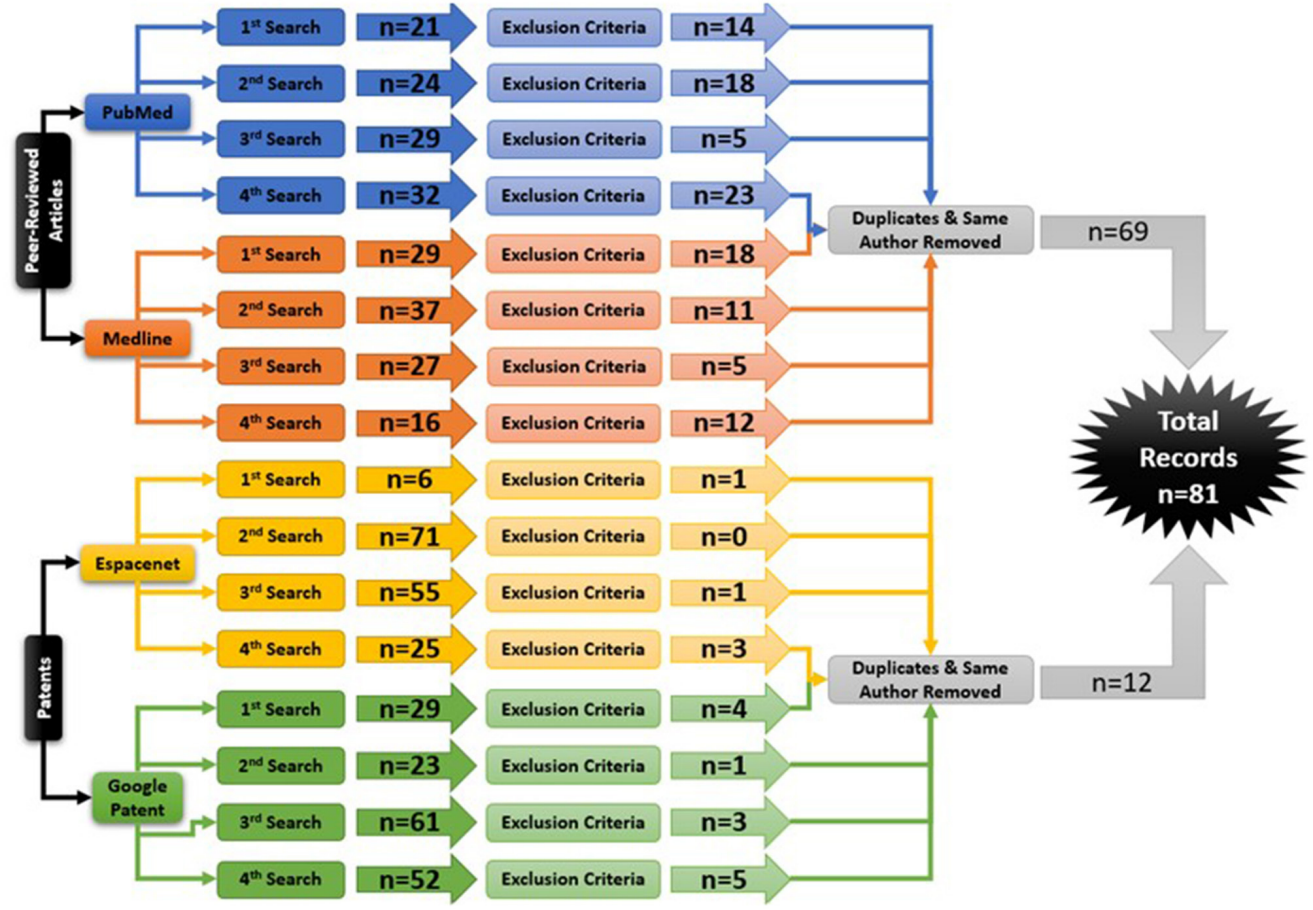
The Hand-Worn Device Review literature selection process is shown in this flow chart. From the original four databases, there were four separate searches done. Each search produced a number of sources that were narrowed down by using the exclusion process. Afterwards, duplicates and same author same device references were removed. This led to a total of 81 references in this paper.

**Figure 4:**
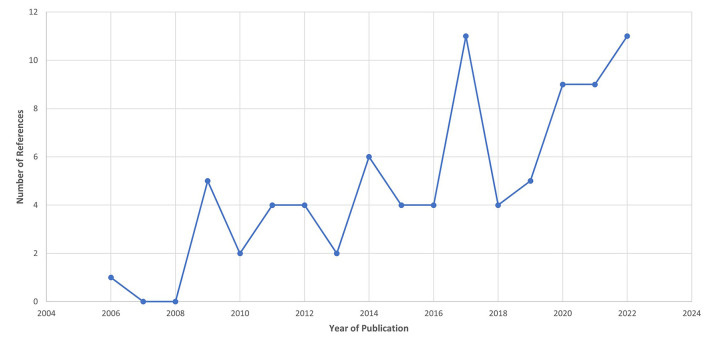
Publication year for the references from the Hand-Worn Device Review show that the majority of the literature was written in the past decade between 2009 and 2022. There is an increase trend of interest in hand-worn devices, particularly after 2016. This projected trend can be used to predict the growing increase in hand-worn devices for rehabilitation and assessment for individuals with impaired hand function.

**Figure 5:**
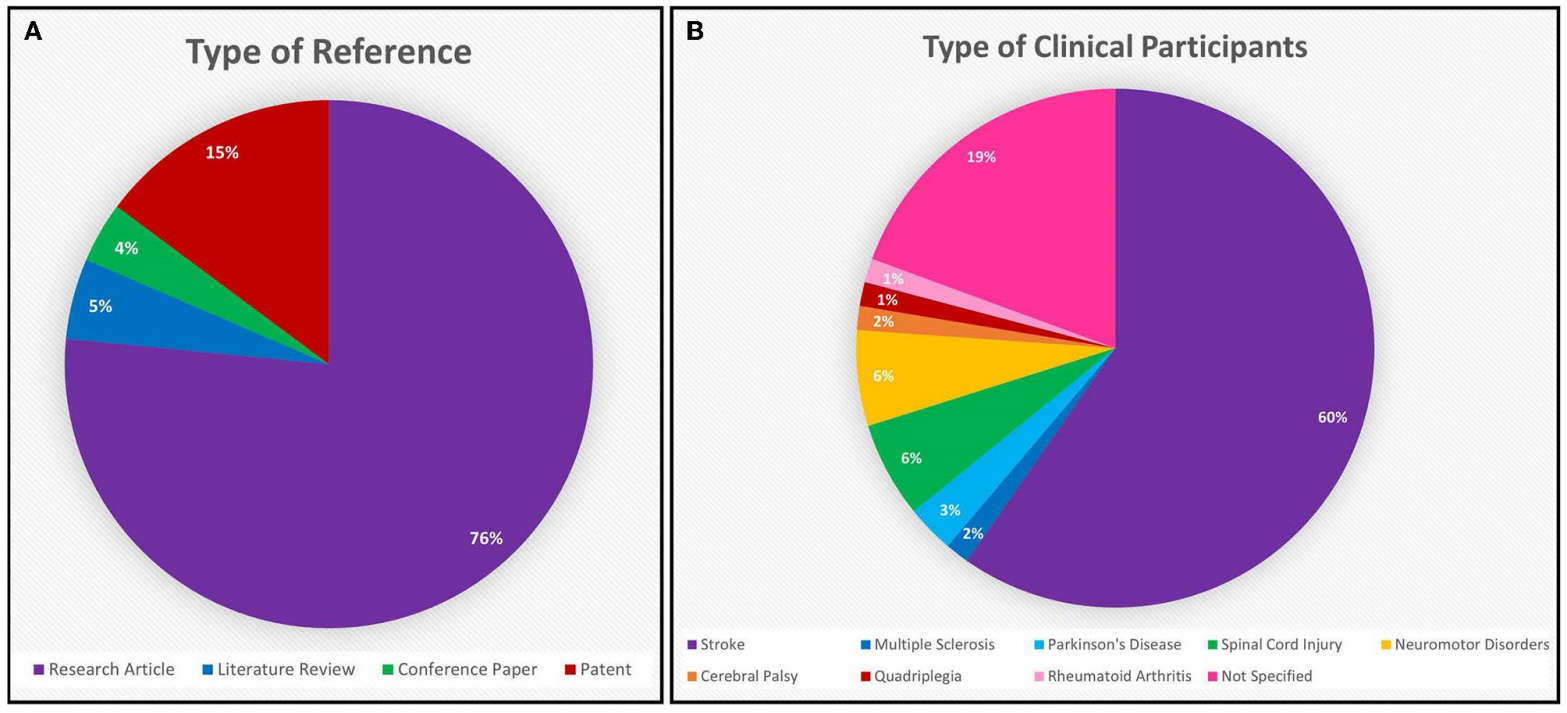
**(A)** The majority of the 81 references from the Hand-Worn Device Review are research articles (76%), while the second leading source is patents (15%). Only 5% of these references are literature reviews that focus on hand-worn devices for rehabilitation and assessment for individuals with impaired hand function. **(B)** The patient population distribution shows that 60% of the population who are targeted to wear these devices are stroke patients. While 19% of these references target any individual who has impaired hand function (given the term: “Not Specified” in the chart).

Following the process described in Sections 2.1 and 2.2 and depicted in Figures 2, 3 (also see Tables 1, 2), 81 references were identified. Further exclusion criteria were applied (see Section 2.3), and duplicate references written by the same group of authors on the same device removed. The majority of the references were published in the past decade, especially between 2017 and 2022 (see Figure 4). The references were sorted into research articles, literature reviews, conference papers, and patents (see Figure 5A); most were research articles and only 15% were patents. These articles were further separated based on whether a clinical study was mentioned. Sixty-two references had clinical components and were further divided by patient profile, the most common being individuals affected by a stroke, who make up sixty percent of the clinical participant population from all the references combined. In Figure 5B, there is a “Not Specified” category for type of clinical participants, which means that a study was conducted with a device developed for clinical use, but the targeted ailment or condition was not specified. Patents were far more likely to not specify their participant profile.

#### 3.1.2. Types of devices

**Figure 6:**
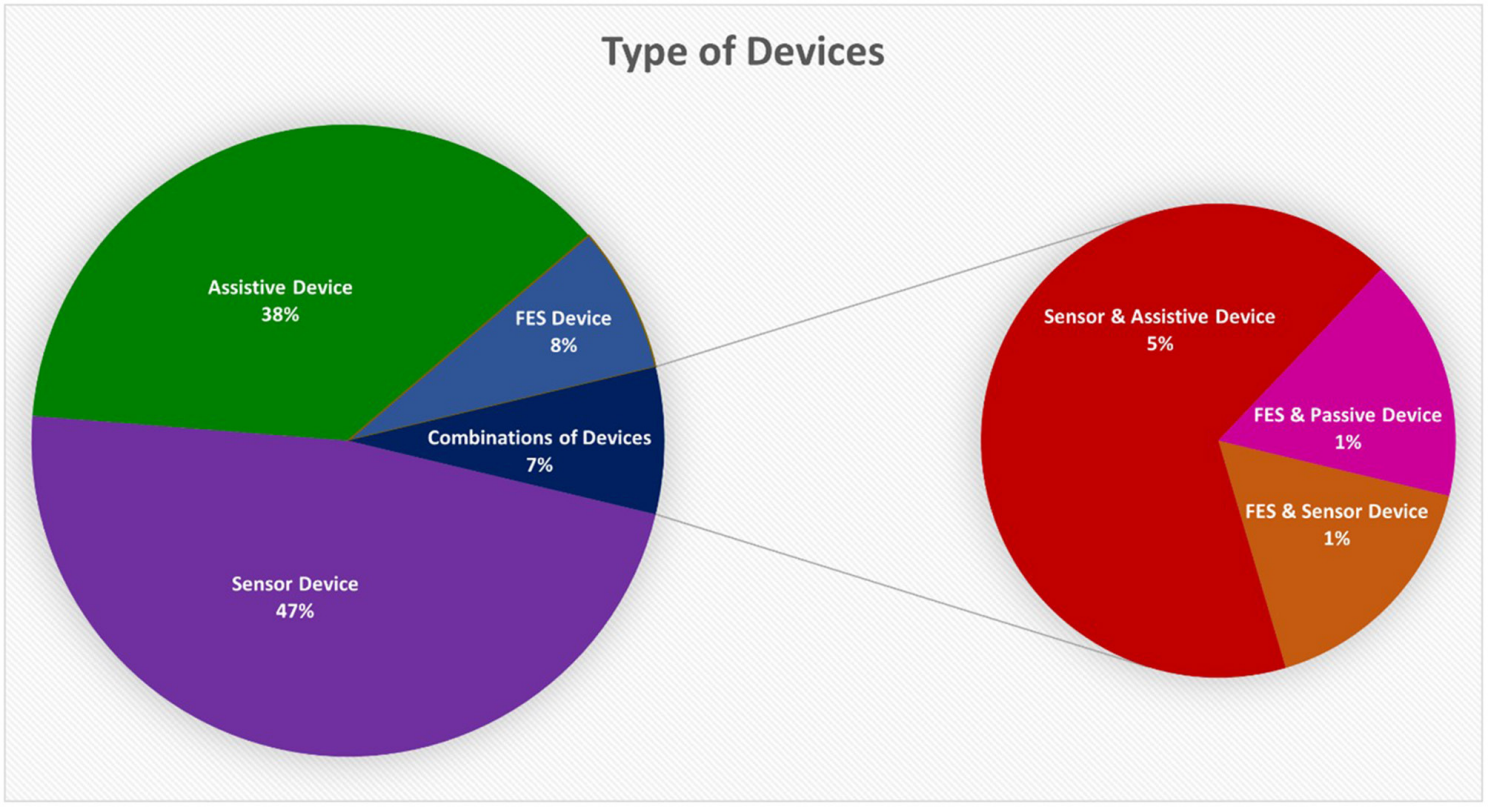
The distribution of the type of device found in all references from the Hand-Worn Device Review showed that sensor-based devices and assistive devices were the most researched or tested. Sensor-based devices make up almost half of the references, while assistive devices make up 38% of the references. FES devices only make up 8% of the 81 references. The smaller pie chart to the right shows the breakdown of all the device combinations from the 7% of the 81 references found in the Hand-Worn Device Review.

Only references related to devices that interact with the hand or incorporate a hand-worn device that either monitors, assists, stimulates, or supports hand function were considered. While there are devices that strictly belong in one of the three major categories (i.e., *Sensor-based, Assistive, FES*), some used a *combination of these devices*. Sensor-based and assistive devices were the most widely documented (see Figure 6). Sensor-based devices were used for functional assessment of the hand or in rehabilitation whereas assistive and FES devices by themselves were used to augment rehabilitation but without the ability to monitor hand function.

##### 3.1.2.1. Sensor-based devices

**Figure 7:**
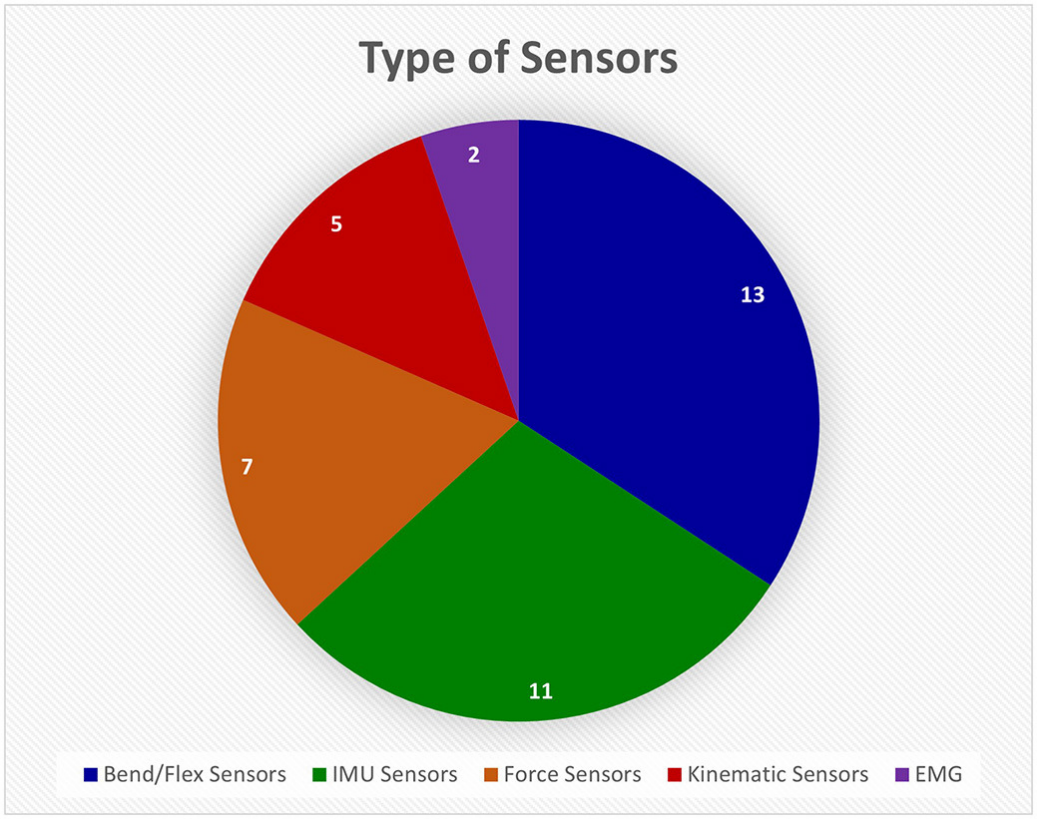
This pie chart shows how many different devices from the Hand-Worn Device Review used a particular sensor type. The most used type of sensor was flex or bend sensors that measure the amount the finger curls. The Inertial Measurement Unit (IMU) sensors were the second most used sensors. The least used sensor was the electromyography (EMG) sensors. The total amount of unique sensors devices was found to be 38. References that use the same device were only counted once in the pie chart.

Most references developed or used sensor gloves in their studies. Sensor-based devices is a very broad category since many sensors can be used to monitor hand function. The different types are broken down in Figure 7. Bend/flex sensors and inertial measurement unit (IMU) sensors are the standard for monitoring movement and relative location because they are inexpensive and easily available. They also produce measurements that can be monitored in real time.

Resistive bend sensors are employed in the commercially available “Cyberglove” device (CyberGlove Systems LLC, San Jose, CA) (Merians et al., 2009; Lee, 2014; Thielbar et al., 2014; Dimbwadyo-Terrer et al., 2016; Ranganathan, 2017; Jarque-Bou et al., 2020; Padilla-Magana et al., 2022). The CyberGlove monitors all individual finger joints. Other commercially available gloves are the P5 Glove (Sivak et al., 2009) (Mindflux, Australia) and 5DT Sensor Glove (5DT Technologies) (Golomb et al., 2010). While these gloves are effective at monitoring hand function, they can be relatively expensive (USD 190 to 2,500 or more) compared to standard video monitoring systems, which may limit their use in at-home rehabilitation (Merians et al., 2009; Sivak et al., 2009; Golomb et al., 2010; Lee, 2014; Thielbar et al., 2014; Dimbwadyo-Terrer et al., 2016; Ranganathan, 2017; Jarque-Bou et al., 2020; Padilla-Magana et al., 2022). Besides commercial bend/flex sensing devices, there are custom devices that address limitations of commercial gloves (Gentner and Classen, 2009; Knutson et al., 2009; Wille et al., 2009; Oess et al., 2012; Jung et al., 2017; Fu et al., 2019; Huynh et al., 2019; Burns et al., 2021; Kim et al., 2022; Syeda et al., 2022). For example, Oess et al. (2010, 2012) developed the NeuroAssess Glove, which has four bend sensors (Flexpoint Sensor Systems, Inc., Draper, UT) to monitor the individual phalanges of the index finger and thumb. The sensors were accurate to within 3 degrees (95% confidence interval) (Oess et al., 2010). Some groups are combining other sensors with bend/flex sensors (Wille et al., 2009; Burns et al., 2021). For example, Wille et al. (2009) custom-designed gloves with bend sensors, accelerometers, and magnetometers placed on the forearm and individual fingers. The glove was meant to be integrated into a virtual reality game to treat children with neuromuscular disorders. A limitation is that the glove is designed for simplicity rather than accurate tracking of movement (Wille et al., 2009). From the reviewed articles, the bend/flex sensors were found widely in research and commercial products. The commercially available gloves are costly, while the gloves designed by research groups have not been fully validated and ready for clinical use.

IMU sensors are also very common in research studies and in commercial gloves (Cavallo et al., 2013; Li et al., 2016; Lemos et al., 2017; Mohan et al., 2018; Salchow-Hommen et al., 2019; Bhagubai et al., 2021; Fei et al., 2021; Kamockij and Fedorov, 2021; Pan et al., 2021; Schwarz et al., 2021; Hwang et al., 2022). They incorporate gyroscopes, accelerometers, and magnetometers to track the wearer's orientation. In regard to commercial systems, Schwarz et al. (2021) used a motion capture system called Xsens MVN Awinda (Xsens Technologies, Netherlands). Custom-designed IMU gloves have also been developed to improve on commercial gloves (Cavallo et al., 2013; Li et al., 2016; Lemos et al., 2017; Mohan et al., 2018; Salchow-Hommen et al., 2019; Bhagubai et al., 2021; Fei et al., 2021; Kamockij and Fedorov, 2021; Pan et al., 2021; Hwang et al., 2022). For example, the i-Glove has embedded IMU sensors on the wrist, index finger, middle finger, and thumb (Mohan et al., 2018). Fei et al. (2021) developed a data glove system with IMU sensors on the individual finger joints. However, they found that IMU sensors can cause potential errors in sensor-to-segment calibration of location and defining the global frame of reference for the sensors (i.e., the sensors' origin in space) (Fei et al., 2021). While IMU sensors are most commonly used, there are cases where a glove has only one of the three measuring devices, the accelerometer, for instance (Gerber et al., 2016). Additionally, some kinematic sensors track spatial and temporal variables (i.e., position, velocity, etc.) of finger and hand movement (Wille et al., 2009; Avanzino et al., 2011; King et al., 2011; Bonzano et al., 2013; Gerber et al., 2016; Bisio et al., 2017; Jung et al., 2017; Adams et al., 2019; Bonassi et al., 2020). The most common system used was the Glove Analyzer System (GAS) (Avanzino et al., 2011; Bonzano et al., 2013; Bisio et al., 2017; Bonassi et al., 2020). GAS attaches these sensors onto the individual finger joints. All the articles that used the device were focused on intervention or hand rehabilitation at home in which this glove was used to monitor physical movement. No specific limitations of the glove were noted, but they are not one-size-fits-all, which means that the end user must select the closest size to their hand. In general, these kinematic sensor measurements are meant to track the velocity of finger movement during a given task (Avanzino et al., 2011; Bonzano et al., 2013; Bisio et al., 2017; Bonassi et al., 2020). From the reviewed references, the IMU sensors were found to be commonly used in research and commercial systems, specifically for motion capture systems. IMU sensors were found to have errors during calibration due to external magnetic fields, therefore they are not very reliable. Similar to the flex/bend sensors, the commercially available gloves are very costly, and the ones currently being developed in research have not undergone extensive testing for validation.

Another noteworthy sensor is the force sensor, which measures the cumulative normal force applied to its surface (Friedman et al., 2014; Signori et al., 2017; Wachter et al., 2018; Wolbrecht et al., 2018; Mawase et al., 2020; Burns et al., 2021; Patane et al., 2022; Urone et al., 2022). In the ergonomic device designed by Mawase et al. (2020), isometric force sensors are placed on the fingertips including the thumb. This was used to test the importance of two different finger functions—maximal voluntary contraction force and individuation (i.e., applied pressure at each finger). They found that rehabilitation of the flexor synergies (i.e., spasticity) could be meaningful in stroke patients' hand function recovery (Mawase et al., 2020). Force sensors are important for determining grip force of the fingers, a key component of overall hand function. However, they have not been widely incorporated into hand worn devices in research and especially in commercially available devices.

The last type of sensor, the electromyography (EMG) electrode, which measures electrical potential changes associated with muscle contraction, was only mentioned in two references per our search criteria as part of a hand-worn sensor glove (Linderman and Rupasov, 2012; Pan et al., 2021). One study used EMG electrodes in a sensor glove to monitor activity in three muscles of the hand. The glove was designed for measurements to be recorded, synced, and stored with EEG for biometric assessments. These assessments focus on showing the correlation between predicted effort (motor imagery (MI) or volitional effort) and physical movement. The importance of this type of system is to show the wearer that even though they may have minimal physical motor function, their effort is measurable and can be harnessed. This EMG sensor glove was the only such device found to be integrated with a BCI system (Linderman and Rupasov, 2012). The limitation with using EMG as a sensor is the difficulty of predicting fine movements of the fingers. This review found that EMG sensors are rarely applied to the hand muscles for monitoring finger movement during muscle contraction.

Overall, sensor-based gloves were used to monitor hand function and provide feedback on the physical movement or applied pressure of the hand. These devices can be used for functional assessment to determine the severity of the impairment or to track a patient's progress through rehabilitation. Due to the simplicity of these devices, they can be integrated into a game (e.g., VR rehabilitation games) to give positive feedback to the patient and motivate them to continue their rehabilitation as well.

##### 3.1.2.2. Assistive devices

**Figure 8:**
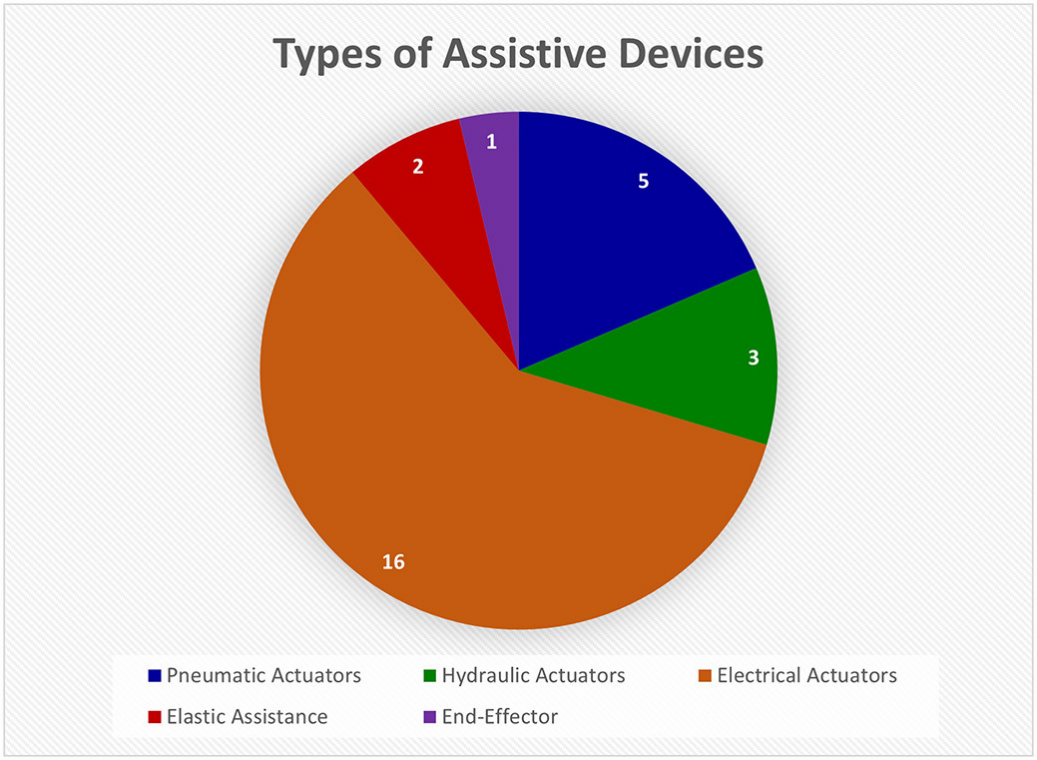
This pie chart shows the relative numbers of articles found on different types of assistive devices in the Hand-Worn Device Review. The most common assistive instrumentation was the electrical actuators followed by the pneumatic actuators. These actuators are attached to the fingers to aid in extension and contraction. The total number of unique devices was 27. Articles that referred to the same device were only counted once.

Assistive devices were the second most investigated type of device and fell into five categories: pneumatic actuators, hydraulic actuators, electrical actuators, elastic effectors, and end-effectors (refer to Figure 8). Electrical actuators appeared to be the most used component of assistive devices (de Araujo et al., 2011; Triandafilou et al., 2011; Iwamuro et al., 2015; Sallum et al., 2015; Fischer et al., 2016; Thielbar et al., 2017; Bernocchi et al., 2018; Chen et al., 2020; Leuthardt et al., 2020; Osuagwu et al., 2020; Yurkewich et al., 2020; Crema et al., 2022; Guo et al., 2022; Luhmann and Randazzo, 2022). A couple of references used a version of the commercially available GloReha glove (Gloreha IDROGENET, Italy), which uses electrical actuators to move individual finger joints. These gloves were used in interventions for at-home hand rehabilitation (Bernocchi et al., 2018) and FES treatment (Crema et al., 2022). Although these studies showed that the interventions improved impaired hand function, there are key limitations. The GloReha glove cannot be worn by patients with severe hand spasticity; and the motor tended to fail if used for long periods (Bernocchi et al., 2018; Crema et al., 2022). An example with non-commercial electrical actuators is the device made by Leuthardt et al. (2020), meant to be used with a BCI to track rehabilitation progress. However, the fingers were actuated in pairs tied together (i.e., index and middle, ring and pinky) and not individually (Leuthardt et al., 2020). Although electrical actuators are commonly used, they are limited in their ability to assist with natural finger movements.

Pneumatic actuators were also commonly used in assistive devices for the hand (Coffey et al., 2014; Thielbar et al., 2014; Yap et al., 2016; Khallaf et al., 2017; Cheng et al., 2020; Jiryaei et al., 2021). A common example is the pneumatic exercise glove (PneuGlove), which induces movement of the whole hand (Coffey et al., 2014; Thielbar et al., 2014). Inflatable bladders hyperextend the hand and induce dorsiflexion of the wrist. Although the glove is comfortable, portable, adjustable and does not obstruct movement, it cannot control individual fingers, only all of them together (Coffey et al., 2014; Thielbar et al., 2014). Other pneumatic actuator devices have been developed for rehabilitation (Cheng et al., 2020; Jiryaei et al., 2021). Cheng et al. (2020) used air pressure actuators in a BCI-based soft robotic glove that assists individual finger movements excluding the thumb. Their protocol used BCI-assisted motor imagery in robotic rehabilitation. They compared the use of the soft robotic glove to assist movement with and without a BCI in the loop. However, neurotypical individuals without hand impairment were not assessed to provide a performance baseline (Cheng et al., 2020). While pneumatic actuators were found to be useful in rehabilitation strategies, they have not been thoroughly validated with a healthy control group or generalized to a range of stroke acuities.

Other assistive devices include hydraulic actuators (Gallo et al., 2017; Yeow et al., 2019; Wijesundara et al., 2021) and elastic effectors (Biggar and Yao, 2016; Lieber et al., 2022). The hydraulic actuators can mimic the articulation of the human hand (Wijesundara et al., 2021). They have also been used in feedback loops. For example, Gallo et al. (2017) developed a multimodal haptic device that uses hydraulic actuators on the individual fingers and feedback sensors on the fingertips. The hydraulic actuators would react to the input modalities (e.g., specific hand postures) and move the fingers accordingly; but the device is bulky, which can restrict movement (Gallo et al., 2017). The elastic assistive devices use elastic bands (Biggar and Yao, 2016) to extend the fingers. The main issue with these devices is that they alter natural grasp configurations. The main finding was that the hydraulic actuators were found to be similar to the electrical and pneumatic actuators but restrict natural hand movement more. The elastic assistive devices were even more constricting to gross hand movement and therefore, are limited in the amount of assistance they can provide to the individual with impaired hand function.

Lastly, there are end-effector devices, of which only one example was found. Zbytniewska et al. (2021) used an end-effector robot for the index finger alone, which is only one degree of freedom (DOF). As mentioned previously, the human hand and wrist have 21 DOF, which means that rehabilitation of all finger movements will be very time-consuming with this one-DOF end-effector robot (Jones and Lederman, 2006; Zbytniewska et al., 2021). This review found confirmation that the end-effector devices are restricted on how much assistance they can provide to an individual's hand/finger movement.

In summary, the assistive devices reviewed were used for rehabilitation to aid in execution of movement. They can provide physical feedback to the patient based on induced and self-initiated movement. Induced movement involves a device physically moving the fingers rather than by stimulation to produce a muscle contraction. These devices were helpful in improving finger and/or hand function.

##### 3.1.2.3. Functional electrical stimulation devices

As shown in Figure 6, FES devices make up 8% of the references (Golaszewski et al., 2012; Krukowska et al., 2014; Lin et al., 2014; Sullivan et al., 2015; Kattenstroth et al., 2018; Friedenberg et al., 2022). They were commonly used in rehabilitation and several clinical studies tested their capabilities in treatments for hand impairments. A common hand-worn FES device is the Mesh Glove (MG), which stimulates muscles over the whole hand (Golaszewski et al., 2012; Lin et al., 2014). The MG was used in interventions with mirror therapy (Lin et al., 2014) and one group aimed to test the glove's utility in rehabilitation (Golaszewski et al., 2012). Another version of FES glove only targeted hand extensor muscles (i.e., three muscles on the hand). This group, Krukowska et al. (2014) compared their glove with standard bipolar surface electrodes placed on the forearm. They found that their device helped restore motor function in the forearm as well as the hand (Krukowska et al., 2014). Another group tested the usability of their FES glove, which delivered stimulation to the hand via embedded electrodes, on chronic stroke patients. It was found difficult to use when completing the intervention tasks (Sullivan et al., 2015). Another group developed a glove that used 20 Hz electrical stimulation from built-in electrodes to target individual fingers rather than the whole hand. Kattenstroth et al.'s (2018) study tested the improvements in hand motor function after a three-and-a-half-week intervention with and without this glove and found it to be more effective than standard therapies. These studies were tested only on mild to moderate, but not severe hand impairments. Therefore, they cannot be used to help the severely impaired individuals.

Overall, FES devices were found to be beneficial in rehabilitation for individuals who have impaired hand function. A lot of these devices used triggers (e.g., unimpaired hand, protocol cues) to stimulate the impaired hand at the appropriate time. FES devices can be paired with conventional therapies (e.g., mirror therapy) or customized rehabilitation protocols.

##### 3.1.2.4. Device combinations

Combinations of the three major device types were as common as FES devices alone (refer to Figure 6) (Merians et al., 2009; de Araujo et al., 2011; Thielbar et al., 2014; Gallo et al., 2017; Hoffman et al., 2017; Wolbrecht et al., 2018; Huynh et al., 2019; Chen et al., 2020; Osuagwu et al., 2020; Kim et al., 2022). Most of these combine sensors and assistive devices because they typically use the former in a glove to control the latter. For example, Kim et al. (2022) used a glove with bend sensors and a 4 DOF soft robotic glove focused on the thumb and index finger. A sensor glove worn on the unimpaired hand was used to help rehabilitate the impaired hand wearing a soft robotic assistive glove (Kim et al., 2022). Another combination involved FES and a sensor-based device, which provided visual feedback to the participant on FES-triggered hand function. Fu et al. (2019) used a kinematic sensor glove for the unimpaired hand, biphasic current-controlled transcutaneous stimulator for the FES, and a fingerless mitten with bend sensors for the impaired hand (Handana Corp., Austin, TX). The sensor glove was used to provide input movement to the FES stimulator. The fingerless mitten sent the impaired hand's movement to a virtual game for rehabilitation. In this way, self-administered FES treatment and video-gaming was possible for a less impaired individual (Fu et al., 2019).

Additionally, Knutson et al. (2009) developed a custom-built command glove with bend sensors on the thumb, index, and middle fingers. The glove was designed for monitoring movement during an FES intervention. The limitation to this device was that it only monitored three fingers and not the whole hand. Therefore, this glove does not provide a complete characterization of hand function (Knutson et al., 2009). The last combination involves FES and passive devices. Passive devices are only used to support the upper extremity but do not facilitate or monitor hand function in any way. In this review, passive devices appeared in one reference: Uswatte et al. (2006) described two different devices—hand splint and fingerless gloves. Their study investigated the effects of different types of training and restraints on recovery of the impaired hand. They found that using or not using these supportive devices made no difference (Uswatte et al., 2006). In the context of this device combination, passive devices provide support to the hand, while the individual is subject to FES intervention. For example, Hoffman et al. (2017) developed an orthotic device with support sections and electrodes on the forearm, hand, and fingers. The limitation of this combination is that the glove cannot monitor physical responses to the electrical stimulation.

In summary, these device combinations were proven useful in their targeted goals (i.e., assisting with movement, etc.). Majority of these combinations used a sensor glove as an input and either a FES or an assistive device as the movement initiator. These system can be very useful in motivating and providing proprioceptive feedback to the user since their unimpaired hand is used to trigger the assistive or FES device on their unimpaired hand.

In addition to combinations of the main type of devices (e.g., sensor and assistive) presented in this review, there were three instances in the literature of combinations of devices integrated into a BCI system (Linderman and Rupasov, 2012; Leuthardt et al., 2020; Guo et al., 2022). Guo et al. (2022) used an assistive device with pneumatic actuators to move the fingers while the subject's EEG was monitored. Particular features detected on the scalp over the occipital lobe when an image was shown to the patient would trigger closing and opening of fingers of the hand by pneumatic actuators. After training with the system, the patients' clinical assessment scores were found to improve significantly (Guo et al., 2022). This BCI-assistive system focused on extracting EEG features over the occipital lobe and not the premotor and primary motor cortex. This is an unconventional choice because it focuses on visual attention—specifically steady-state visual evoked potentials (SSVEPs)—rather than motor pathways associated with hand function. Guo et al.'s (2022) system requires little training and targets different neurological mechanisms from MI or volitional controlled BCIs. As mentioned in the introduction, the premotor and primary motor cortex are responsible for volitional control of hand movement. The only reference that used a sensor glove with EEG was by Linderman and Rupasov (2012). They developed an EMG sensor glove that monitored activity in three muscles of the hand. They synced the EMG and EEG signals to detect potential biomarkers of Parkinson's Disease (PD). Using only three channels of EMG can limit the ability to detect the PD biomarkers in physical finger movement (Linderman and Rupasov, 2012). These references found that using brain activity to be integrated with a hand-worn device was beneficial to the patient by improving motor function and gain vital information on the relationship between finer hand movements and the brain. These types of studies lay the foundation for BCI development.

Overall, these device combinations proved effective in rehabilitation interventions. The sensor-based gloves can be used to provide input (i.e., the trigger or controller) to an assistive device, an FES device, or a monitoring device, and to confirm movement or applied pressure. This dual utility of sensor-based gloves allows them to aid in both rehabilitation and assessment of hand function. The FES and assistive devices can only be used for rehabilitation when not paired with a sensor-based device. The integration of these devices with EEG was also found to be helpful in both assessment—when paired with a sensor-based device—and rehabilitation. All of these devices have been used in motivational therapies (i.e., games) to provide feedback to the wearer in either assisting with activities or confirming that the user completed specific tasks.

### 3.2. Review of hand-worn devices used with BCIs

#### 3.2.1. Study selection, characteristics, and populations

**Figure 9:**
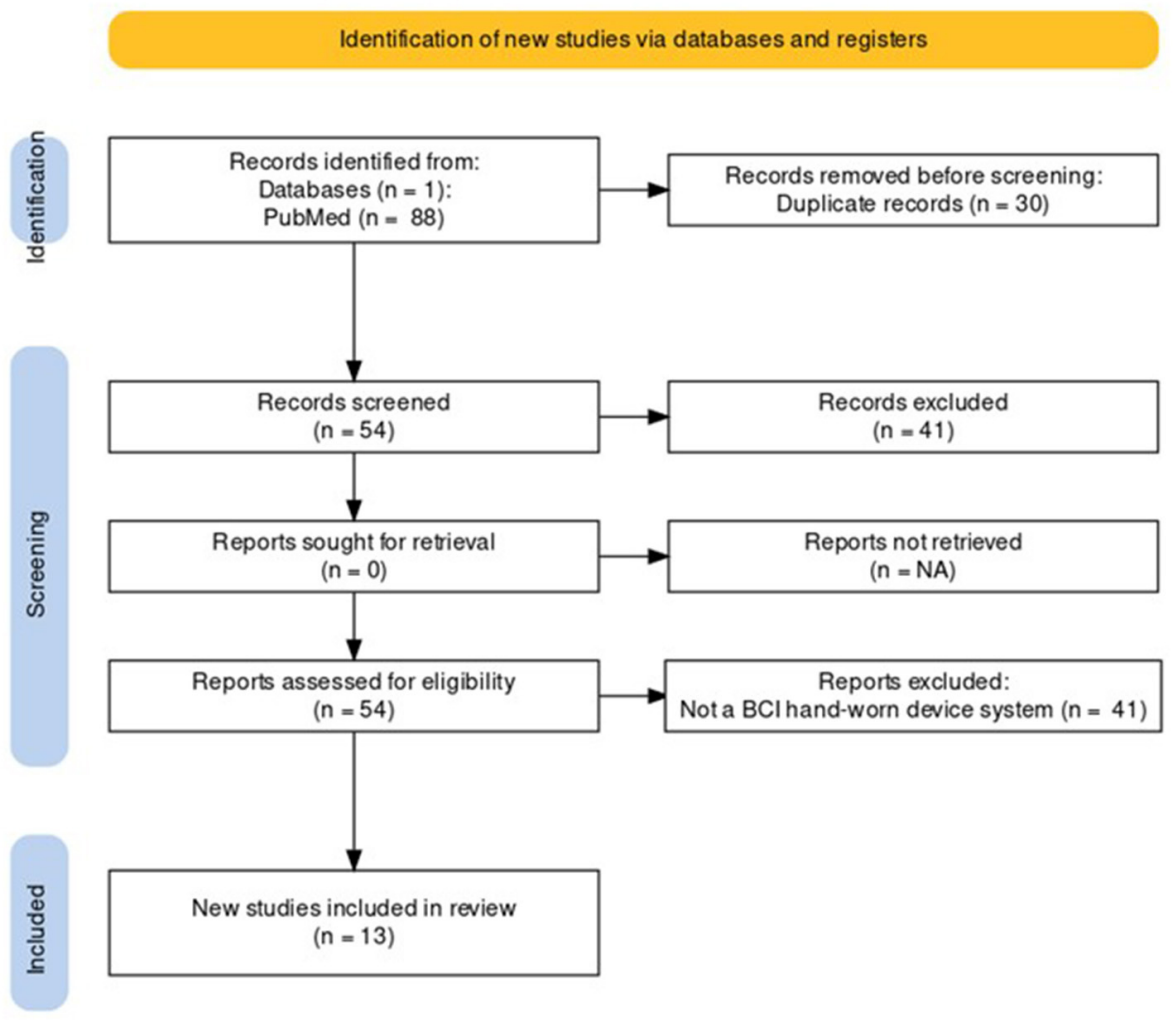
PRISMA flowchart for the BCI Hand-Worn Device Sub-Review for rehabilitation and functional assessment for individuals suffering from impaired hand function. This review conducted four different searches for literature (i.e., research articles, clinical trials, control trials, other reviews) that focus on BCI hand-worn devices. From the original 54 references found, only 13 of those references remained after exclusion criteria (i.e., must be a device attached to the hand) were applied.

**Figure 10:**
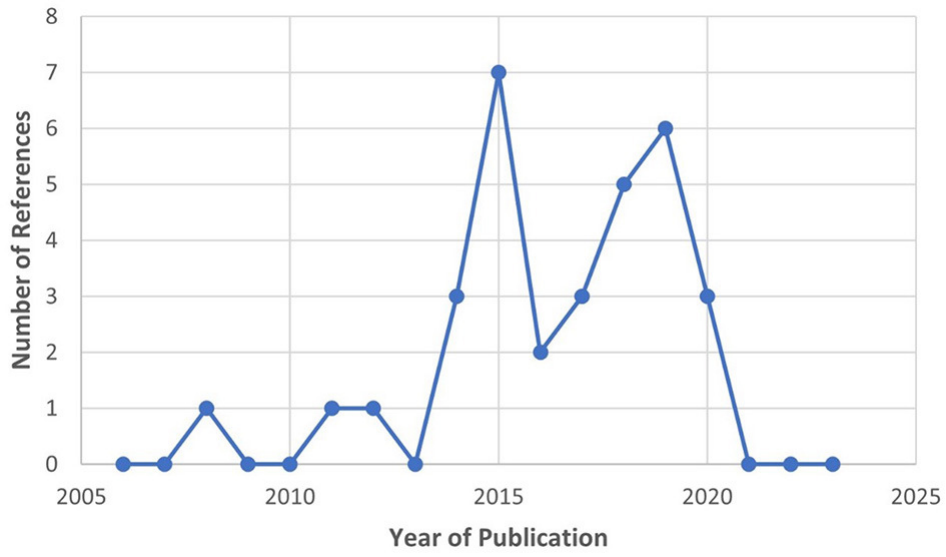
Publication year for the extracted research articles from the 13 BCI Hand-Worn Device Sub-Review, which shows that the majority of the literature was written between 2014 and 2020. Important to note that there was a decrease in interest after 2019.

The selection process of the literature is summarized in Figure 9. Thirteen references were identified through the PubMed searches (see Table 3). After these searches, further exclusions were applied (refer to Section 2.3) and duplicates were removed. Most of the references are recent, especially between 2020 and 2022. They were sorted by type of article (i.e., research articles and literature reviews). The majority turned out to be literatures reviews on Assistive BCI systems. Due to the large quantity of review articles, the authors combined all the review articles' (Cervera et al., 2018; Bai et al., 2020; Kruse et al., 2020; Baniqued et al., 2021; Behboodi et al., 2022; Mansour et al., 2022; Peng et al., 2022; Xie et al., 2022) studies/reports. The eight literature reviews produced 32 unique research articles that were within the scope of this sub-review, and they are described in Supplementary Table V. Majority of these research articles were published between 2014 and 2020 (refer to Figure 10). As mentioned in Section 2.5, the table identifies key characteristics of the BCI hand-worn device system used in the research articles' study: the hand-worn device, BAM device, the studies' purpose, results, and the limitations of the hand-worn device. These systems were found to be used for clinical applications only and they mainly targeted the stroke patient population.

#### 3.2.2. Types of BCI systems

**Figure 11:**
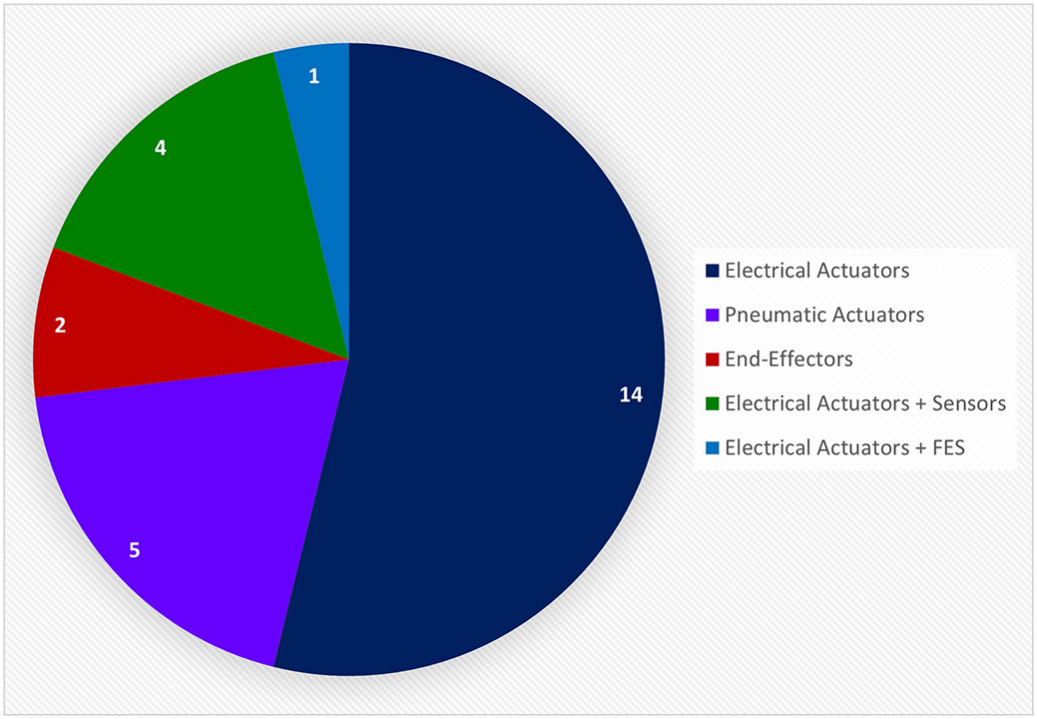
This pie chart shows the number of unique devices used in the relative research articles extracted from the BCI Hand-Worn Device Sub-Review. Majority of the research articles used electrical actuators in the form of an exoskeleton or orthosis as the proprioceptive feedback in their BCI system. None of these systems included only a sensor-based device to provide accurate monitoring and confirmation of hand movement. There were, however, four systems that used electrical actuators with sensor-based devices. There was a total of 26 unique systems from the 32 research articles extracted from the 13 references. Articles that referred to the same device were only counted once.

**Figure 12:**
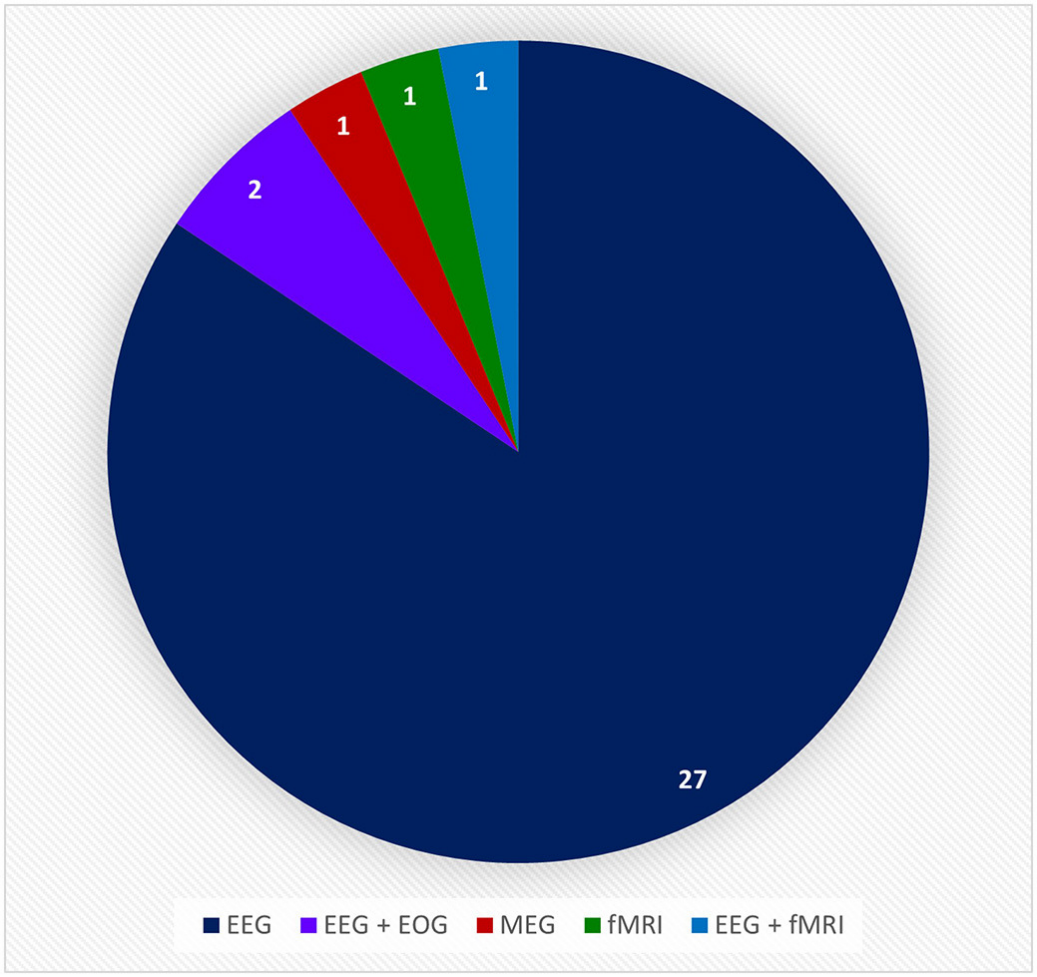
This pie chart shows the number of times a particular brain activity monitoring device was used in the relative research articles from the BCI Hand-Worn Device Sub-Review. The majority of the research groups (i.e., 27 out of 32 articles) used EEG as the brain activity monitoring (BAM) device for their BCI system. EEG, electroencephalogram; ECOG, electrocorticography; MEG, magnetoencephalography; EOG, electro-oculography; fMRI, functional magnetic resonance imaging.

In this review, only the 32 references that incorporate a hand-worn device with a BAM device were included. These in turn were initially of three types: *Assistive, Assistive* + *Sensor-based*, and *Assistive* + *FES*. Instances of FES devices used in conjunction with a BAM were excluded though since the FES was never worn on the hand or as a glove, which is the main focus of this review. Devices were further broken down by the mechanism of assistance, which were of five categories: *Electrical Actuators, Pneumatic Actuators, End-Effectors, Electrical Actuators* + *Sensors*, and *Electrical Actuators* + *FES*. All of these hand-worn device instrumentations were described in Section 3.1.2. Figure 11 depicts the number of unique hand-worn devices found in the 32 articles. The same device used in different research articles was only counted once. *Electrical Actuators* were most commonly used in BCIs. Additionally, articles were categorized based on the BAM method: *EEG, EEG* + *EOG, MEG, fMRI*, and *EEG* + *fMRI*. As shown in Figure 12, EEG was the most common BAM method used in a BCI system.

Most of these articles tested the feasibility of their BCI in stroke rehabilitation (Buch et al., 2008; King et al., 2011; Holmes et al., 2012; Coffey et al., 2014; Witkowski et al., 2014; Barsotti et al., 2015; Cantillo-Negrete et al., 2015; Kasashima-Shindo et al., 2015; Stan et al., 2015; Bundy et al., 2017; Nishimoto et al., 2018; Norman et al., 2018; Ono et al., 2018; Randazzo et al., 2018; Li et al., 2019; Zhang et al., 2019; Chowdhury et al., 2020). For example, Barsotti et al. used a BRAVO exoskeleton, which uses 5 planar electrical actuators driven by two motors—one for the thumb and the other for the rest of the fingers—and EEG. They tested this BCI's feasibility in helping stroke patients perform self-initiated grasping movements. They found that incorporating a BAM device with an assistive device can allow patients to exert volitional control over the assistance. A limitation with their study however is that they did not evaluate the performance metrics from the system during the exercise (Barsotti et al., 2015). Another feasibility study demonstrated the FINGER Robotic Exoskeleton, which extends one finger at a time while inhibiting the movement of the other fingers, with EEG control to allow for more complex movements (Norman et al., 2018). They showed that this BCI system can be a promising tool to enhance motor function recovery, but their exoskeleton only attaches to two fingers at a time (Norman et al., 2018). Both of these studies mentioned the use of EEG only, but there has been some effort devoted to using EEG+EOG as the BAM device (Witkowski et al., 2014). Witkowski et al. (2014) sought to test the hypothesis that integrating EOG into the BAM device will improve the control and safety of the hand exoskeleton. They used the BioRobotics Institute Hand Exoskeleton (Scuola Superiore Sant'Anna, Pisa, Italy) to assist with index finger and thumb movement, which only allows a pinching motion. Nevertheless, they concluded that a hybrid EEG/EOG BCI-controlled exoskeleton can improve the effectiveness of training protocols (Witkowski et al., 2014). Additionally, some groups looked into what area of rehabilitation (e.g., assistive, prosthetics, etc.) their device could benefit. Ono et al. (2018) used the Power Assist Hand (Team ATOM, Atsugi, Japan), which has pneumatic actuators to assist with extension and flexion of fingers, with one EEG channel on either C3 or C4 (depending on the hand being used). The Power Assist Hand was programmed to open and close the fingers in sync and therefore did not assist individual finger movement. They found that adding realistic proprioceptive feedback using event-related desynchronization (ERD) power fluctuations associated with motor imagery (MI) was beneficial to rehabilitate patients' hand function (Ono et al., 2018). All of these references mentioned here were testing the feasibility of their BCI hand-worn device system in a rehabilitation setting for individuals with impaired hand function. They found that their systems were proven effective in their purposed goal, however majority of these hand-worn devices were not designed to assist with individual finger movements of the whole hand.

Other studies in this sub-review compared their BCI rehabilitation systems against traditional therapies (Ang et al., 2014; Naros et al., 2016; Frolov et al., 2017; Ramos-Murguialday et al., 2019; Tsuchimoto et al., 2019; Wada et al., 2019; Cheng et al., 2020). For example, Cheng et al. (2020) tested a BCI-controlled hand exoskeleton—a Soft Robotic Module with 4 pneumatic actuators and a thumb splint—and found improvements in motor function compared to standard methods. A potential drawback to this design is that the splint prohibits movement of the thumb, which can limit assistance to the whole hand (Cheng et al., 2020). Another study by Frolov et al. (2017) compared an EEG BCI exoskeleton with pneumatic actuators on all the fingers against only an assistive device (i.e., exoskeleton) intervention to see the benefit of incorporating the BAM device with the hand-worn device. They found that BCI system was more beneficial in promoting motor recovery compared to passive repetitive movements controlled by the assistive device. This system could extend and contract the fingers together but they did not demonstrate individual finger movement (Frolov et al., 2017). Another example of a group that was testing the benefits of their BCI system compared to traditional therapies was Ang et al. (2014) and they used an End-Effector and EEG. Their End-Effector was a Haptic Knob Robot that was strapped to the hand and controlled by EEG signals. They compared the Fugl-Meyer Motor Assessment (FMMA) scores from their BCI group with the control group, who received traditional therapy and they found that the BCI group achieved significantly higher scores. However, the motor improvements of the FMMA scores are limited by the device because even though it was strapped to the hand, it focuses on proximal arm movement. Therefore, these gains cannot be directly related to ADLs (Ang et al., 2014). All of these articles tested the systems capabilities to improve the standard rehabilitation interventions and strategies. The BCI hand-worn systems were proven effective in promoting motor recovery, which can be seen in clinical assessments preformed by the individuals.

Only a few studies examined brain activity features in relation to BCI intervention with a hand-worn device (Bauer et al., 2015; Ramos-Murguialday and Birbaumer, 2015; Vukelic and Gharabaghi, 2015; Ono et al., 2016; Tacchino et al., 2017; Wang et al., 2018; Carino-Escobar et al., 2019; Caria et al., 2020). For example, Tacchino et al. (2017) used the GloReha glove (Gloreha IDROGENET, Italy) (mentioned in Section 3.1.2.2) to assist hand movement using a robotic device controlled by the participant. They found that having active participation in controlling the assistive device can cause stronger and longer lasting ERD in their brain activity when the patient receives afferent proprioceptive feedback via an assistive device (Tacchino et al., 2017). Another group used a Robotic Hand Orthosis and EEG to investigate how neural plasticity in the motor networks change after BCI intervention in chronic stroke patients who have severely impaired hand function. Their hand-worn device used electrical actuators for assisting with movement and had optical sensors to monitor finger position. Due to the design of the Robotic Hand Orthosis, the wearer cannot form a closed fist because there is a cylinder on the inside of the hand that connects the electrical actuators on the fingers to the rest of the device (Caria et al., 2020). Caria et al. (2020) found that BCI-assistive intervention can not only reinforce ipsilesional brain activity but elicit intra- and inter-hemispheric reorganization with proprioceptive feedback. Additionally, other groups investigated the effectiveness of different types of BCI feedback and whether they promote movement-related brain activity (Vukelic and Gharabaghi, 2015). Vukelic and Gharabaghi (2015) used a commercial end-effector device called the Amadeo Hand Robot (Ectron Ltd, United Kingdom) to control finger movement via mechanical sliders and EEG. The Amadeo Hand Robot restricts natural hand movement because the fingers are strapped to sliders that have to follow a specific linear path. They observed differences in beta-band oscillations depending on whether the individual received visual or proprioceptive feedback. They found that the proprioceptive feedback resulted in lower variability and consistently maintained beta-band oscillations compared to visual feedback (Vukelic and Gharabaghi, 2015). The articles mentioned above focus on gathering more information about how brain activity changes when performing certain actions or completing certain tasks. They have found that using an assistive device with a BAM device can promote neuroplasticity in the brain during intervention and the proprioceptive feedback that the assistive device gives to the patient can prolong motor activity in the brain.

Overall, the BCI hand-worn device systems were proven to be more beneficial in rehabilitation to an individual with hand impairments compared to hand-worn device only interventions or the clinical standard therapies. Not only due these systems provide functional recovery to the individual, but they allow for researchers and clinicians to better understand how the brain reacts to certain stimuli and activity. This is vital in trying to improve neuroplasticity in the brain after injury, which will in turn help recovery hand function.

## 4. Discussion

### 4.1. Summary of main findings

This review found a variety of hand-worn devices used in rehabilitation and assessment of hand function, and examined their limitations. The most common and natural way to attach instruments to the hand is in the form a glove. Gloves allow sensor-based devices to be more accurate because they closely fit the individual finger joints and follow the hand's natural movements. There are many sensor gloves on the market, but not without concerns. For one, they can be prohibitively expensive for the individual and not one-size-fits-all, which means multiple gloves of different size and fit would be needed for research that targets a diverse group of individuals (Merians et al., 2009; Lee, 2014; Thielbar et al., 2014; Dimbwadyo-Terrer et al., 2016; Ranganathan, 2017; Jarque-Bou et al., 2020; Padilla-Magana et al., 2022). They are also not made for convenient home use by individuals with neurological impairments and musculoskeletal disorders (Mohan et al., 2018). The sensors themselves may have quirks such as measurement drift or calibration error. IMU sensors are highly calibration-dependent, and the calibration is usually affected by the regional electromagnetic environment. Therefore, their measurements are frequently off by some percentage of error (Bhagubai et al., 2021; Fei et al., 2021).

Despite these limitations, sensor gloves have great potential for monitoring or functional assessment (Oess et al., 2012; Cavallo et al., 2013; Bhagubai et al., 2021), Combining different types of sensors onto one glove would give a more detailed characterization of hand function. For example, in the Sensoriglove developed by Burns et al. (2021), a flex sensor is attached to the dorsal surface and a force sensor to the tip of each finger. This device was deemed accurate for grasping control. A limitation is that the whole glove has to be replaced if any sensor is damaged during manufacture or degraded by exposure to excessive temperature, vibration, or voltage (Burns et al., 2021). Combinations of IMUs and kinematic sensors have been employed as well. The YouGrabber, designed by Gerber et al. (2016), includes IMUs and accelerometers on the thumb, index, and middle fingers, and was developed for a UE exergame training program. However, wearers found the device to be uncomfortable and the IMUs had calibration errors (Gerber et al., 2016). After reviewing many sensor gloves, we feel that there is a need for an easily fixable, multimodal sensor glove that accurately tracks human hand function. If certain limitations are addressed, such sensor-based devices have great potential for providing objective assessments of hand function during therapy.

Two other literature reviews on assistive devices were found. One is by Balasubramanian et al. (2010) on robot-assisted rehabilitation of hand function. Based on thirty references reviewed, they concluded that simple robotic devices with only one or two specific functions are preferable for at-home hand rehabilitation (Balasubramanian et al., 2010). Another review by Lum et al. (2012), on assistive devices for impaired hand function, found that many groups are developing robotic devices to overcome the challenges of movement-assistive therapy for hand function with multiple degrees of freedom. However, the ability to determine the most appropriate subject population and the exact nature of improvement in hand function with assistive device treatment compared to conventional treatment needs to be investigated carefully (Lum et al., 2012). These reviews were focused on the utility of the assistive devices in clinical applications and briefly touched on the instrumentation flaws within these assistive devices.

Taking these other reviews into consideration, we found that most assistive technologies use actuators to facilitate movement. Many of the references discussed linear actuators and how they were designed to mimic natural hand function with the use of an exoskeleton tendon design (Merians et al., 2009; Triandafilou et al., 2011; Iwamuro et al., 2015; Fischer et al., 2016; Chen et al., 2020; Osuagwu et al., 2020; Yurkewich et al., 2020; Kim et al., 2022). These actuators—whether electric, hydraulic, or pneumatic—do not fully mimic natural human hand functions and may in fact hinder functions of the hand or other parts of the body (Merians et al., 2009; Triandafilou et al., 2011; Oess et al., 2012; Biggar and Yao, 2016; Fischer et al., 2016; Yap et al., 2016; Gallo et al., 2017; Galloway et al., 2019; Yeow et al., 2019; Chen et al., 2020; Guo et al., 2022). Hence, their value in ADLs is debatable. Furthermore, these devices cannot be attached without someone's help. The idea of an assistive device for at-home use is to make a person with impaired hand function more independent, but the devices found in this review still require assistance from a clinician, research, or caregiver to wear and require continuous monitoring. We conclude that while assistive devices have great potential in clinical rehabilitation, there is a need for simpler assistive devices that do not hinder natural human function for assisting in ADL and can be used by severely impaired individuals.

There is still a lot of research to be done on incorporating FES into a hand-worn device. For example, Friedenberg et al. (2022) designed an FES system based on an electrode array that could target any part of the body. However, due to errors in calibration the stimulation was sometimes uncomfortable or painful to the participant (Friedenberg et al., 2022). On the other hand, Kattenstroth et al. (2018) developed a stimulation glove that used built-in electrodes to apply 20 Hz electrical stimulation to the fingertips. This stimulation was to provide tactile feedback and not to initiate movement (Kattenstroth et al., 2018). Both groups mentioned in Section 3.2.3 were able to create a mesh glove that stimulated the hands of mild to moderately impaired individuals. None of these gloves were tested on individuals with severe hand impairments (Golaszewski et al., 2012; Lin et al., 2014). In general, more investigation is needed to set appropriate values for FES stimulation parameters and determine how best to use these devices to help the severely impaired recover hand function.

**Figure 13:**
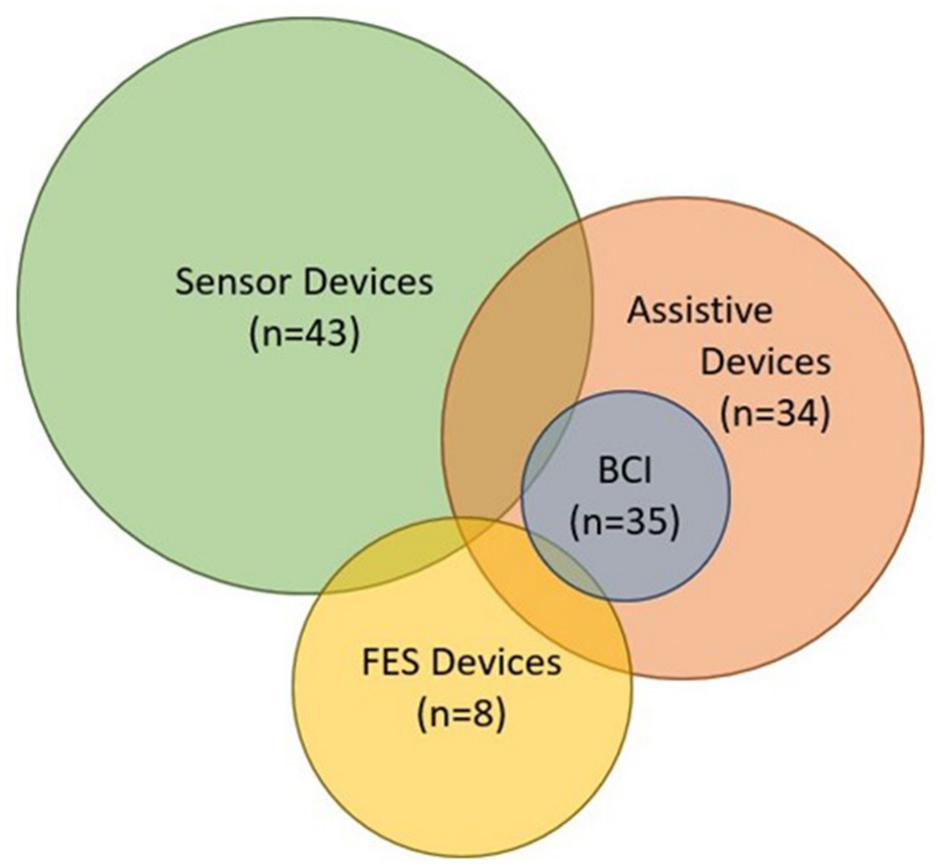
Venn diagram containing all of the devices from the Hand-Worn Device Review and the devices extracted from the BCI Hand-Worn Device Sub-Review. This diagram shows how much each device relates to the others. The size of the circle represents the amount of research and development being done in that particular area. Their relation to each other is shown by the amount of overlap between the circles. The BCI in this diagram stands for the BCI systems that are specifically documented with a hand-worn device. There were more BCI-Assistive systems than BCI-Sensor-based or -FES systems.

Some major aspects of device design for assessment and rehabilitation also need further investigation. Figure 13 shows a Venn diagram of all devices found by this review including those that involve BCIs. The relative size of the circles reflects the volume of literature found for each device type. Overlapping regions represent the amount of effort devoted to integrating multiple types of devices into one system (e.g., device-computer interface or brain-computer interface). A handful of devices used sensor-based devices for input and assistive or FES devices to generate an output in response (Knutson et al., 2009; Fu et al., 2019; Kim et al., 2022). These devices showed great potential for improving rehabilitation strategies when used in a device-computer interface. In contrast, the integration of hand-worn devices into BCI systems appear to be uncommon or sparsely documented. The most common devices integrated with BCIs were assistive (Buch et al., 2008; King et al., 2011; Holmes et al., 2012; Ang et al., 2014; Coffey et al., 2014; Witkowski et al., 2014; Barsotti et al., 2015; Bauer et al., 2015; Cantillo-Negrete et al., 2015; Kasashima-Shindo et al., 2015; Ramos-Murguialday and Birbaumer, 2015; Stan et al., 2015; Vukelic and Gharabaghi, 2015; Naros et al., 2016; Ono et al., 2016, 2018; Bundy et al., 2017; Frolov et al., 2017; Tacchino et al., 2017; Nishimoto et al., 2018; Norman et al., 2018; Randazzo et al., 2018; Wang et al., 2018; Carino-Escobar et al., 2019; Li et al., 2019; Ramos-Murguialday et al., 2019; Tsuchimoto et al., 2019; Wada et al., 2019; Zhang et al., 2019; Caria et al., 2020; Cheng et al., 2020; Chowdhury et al., 2020; Guo et al., 2022). According to Angerhofer et al. (2021), “a major advantage of BCI-based exoskeleton control is that it allows patients to perform grasping movements with their paralyzed hand and enables them to perform bimanual tasks in training sessions”. This ability to exert volitional control over a device that assists with movement is what makes BCI-based assistive systems appealing to researchers, clinicians, and patients. Depending on the studies, either motor imagery or volitional movement was used as the control signal. Other literature reviews have found that volitional temporal or spatial control based on BAM of the hand-worn device increases its functionality and can be more engaging for patients trying to perform manual tasks (Baniqued et al., 2021). Additionally, volitional effort in impaired hand movement followed by real movement (if applicable) can improve motor function more than imagined movement alone (Mansour et al., 2022). This is one of the reasons BCI-assistive systems are more favorable compared to BCI-FES and BCI-sensor-based systems—they promote volitional movement when integrated into a BCI system. Moreover, although they found that FES and robotic movement assistance showed no difference in effectiveness in BCI systems (Behboodi et al., 2022), assistive devices are more likely to target finer finger movement than FES since they can be attached to the hand.

Few devices used a sensor glove as the hand-worn device in a BCI system. As mentioned previously, Linderman and Rupasov (2012) was the only reference that used EEG in tandem with a sensor glove. This sensor glove used EMG to monitor only three muscles, which means finer finger movements may not have been sensed. This is problematic for severely impaired individuals because their movement may be minimal and harder to detect. Therefore, being able to detect somatosensory signals (i.e., information from the skin and from muscle and joint receptors) is key in making successful BCI-based rehabilitation strategies for those with impaired motor function (Pillette et al., 2020). Integrating a sensor glove that monitors fine hand movements as well as applied pressure with BAM device would allow for better characterization of hand and finger function as well as the neural correlates of those movements in the brain.

Overall, BCI systems appear to be safe except perhaps for fatigue and headache associated with exertion (Buch et al., 2008; Ang et al., 2014; Kasashima-Shindo et al., 2015; Bundy et al., 2017; Frolov et al., 2017; Nishimoto et al., 2018; Norman et al., 2018; Carino-Escobar et al., 2019; Ramos-Murguialday et al., 2019; Bai et al., 2020). Additionally, BCI-based hand rehabilitation shows significant improvement in clinical assessment scores compared to other conventional therapies (Cervera et al., 2018). BCIs could help patients with hand impairment through many different stages of recovery. For example, they can be used in the early stages of rehabilitation with BCI-Assistive systems to help with hand movement and functional assessment with BCI-Sensor-Based systems to track progress or diagnose the severity of the impairment. BCIs can even help during the plateau in motor recovery by providing assistance with ADLs at home (Angerhofer et al., 2021). We found in our review that BCI-Assistive systems are most beneficial in neurorehabilitation for hand function tasks. These systems focus on improving neuroplasticity of the individual with impaired hand function by providing proprioceptive feedback (extrinsic stimuli) based on assistive movement of the impaired hand. This feedback is vital in motivating the patient to continue their course of treatment, which will further improve their recovery that can be tracked by a BCI-Sensor-Based system.

As mentioned previously, the BCI-Sensor-Based systems are more useful in tracking or monitoring a person's effort and physical ability to complete a task. They can be used as tools in clinical assessments for objective scoring but can also be incorporated into gamified BCI rehabilitation protocols. Gamifying these BCI systems can promote motivation and positive feedback (Wille et al., 2009). A survey by Ahn et al. (2014), found that creating a team of researchers, developers, and users of BCI games would be beneficial when developing prostheses and improving current rehabilitation strategies. These BCI games would be beneficial in rehabilitative interventions. A couple of references recommend that these interventions should last roughly 4 weeks or longer with high intensity training (approximately five trainings per week) (Kruse et al., 2020; Peng et al., 2022). Due to these intense, lengthy interventions, gamifying the BCI systems will be useful in sustaining the patient's motivation to continue treatment (Ahn et al., 2014). Further research and development into BCIs that incorporate hand-worn devices is vital for enhancing neurorehabilitation in individuals with hand impairments. While assistive devices have been widely studied and developed from being bulky devices to soft gloves, more research is needed to solidify the findings. Therefore, there are existing limitations, which hinder the integration of these BCI systems into rehabilitation protocols. These studies have small sample sizes, short intervention durations, and interpretation difficulty of results due to many factors (e.g., severity of stroke, acuity of stroke, protocol of the intervention, etc.), which leads to a lack of evidence in proving the effectiveness of these systems and discourages medical companies and hospitals from using them in their routine treatments (Mcconnell et al., 2017; Hu et al., 2022; Xie et al., 2022).

### 4.2. Limitations

While conducting this review, we had limited access to certain references. When searching for patents, there was a limitation based on what language the patent was written in. Patents in languages other than English were excluded and translations were difficult to find. Another limitation was that most patents included based on the initial database searches were about a computer system rather than a hand-worn device. For example, some were about a computer program used to control a BAM device and a hand-worn device but did not discuss the hand-worn device itself, only the software component. Additionally, there was limitations regarding information within the articles and patents themselves. We were unable to extract cost; easy of use in general and at home; and time and competences for every device. Some devices were not tested or did not report that information at all. Therefore, we were not able to compare those factors between devices within this review. There is a need to compare these factors with these devices when that information becomes available in future publications.

### 4.3. Conclusions

This review gave an overview of various hand-worn devices and found potential areas that need further exploration. There are three key areas that need to be addressed further:

1. Sensor-based devices need to be easily repairable, affordable, and comfortable. They need to incorporate a combination of sensors to provide a more objective and detailed picture of hand function for clinical functional assessments: one type of sensor alone is not enough to capture the movement and forces on individual fingers.
2. Assistive devices need to be more comfortable and user-friendly without restricting natural movement to be useful in hand rehabilitation protocols and assisting in ADLs.
3. The combination of such devices—particularly sensor gloves—with BAM devices in a BCI system would improve neurorehabilitation protocols. They will help with stimulated as well as volitional hand movement in a clinical setting. Most importantly, these systems will provide proprioceptive, somatosensory feedback to individuals with impaired hand function.

The surge in interest in these devices is evident in this review. There are many avenues that can be researched or further developed to help improve the quality of life for individuals who have impaired hand function caused by neurological trauma (e.g., stroke). Future literature reviews could delve deeper into the areas mentioned above to fill out the picture.

## Data availability statement

The original contributions presented in the study are included in the article/Supplementary material. Further inquiries can be directed to the corresponding author.

## Author contributions

MB and SS conceived the scope of design of the study. MB independently performed a systematic literature review and wrote the first draft of the manuscript. SS performed revisions and provided feedback, which MB used to further refine the manuscript. All authors contributed to manuscript revision, read, and approved the submitted version.
