# Supplementary material for: Hand-worn devices for assessment and rehabilitation of motor function and their potential use in BCI protocols: a review
Source: Front Hum Neurosci. 2023 Jul 6;17:1121481. doi: 10.3389/fnhum.2023.1121481 (PMC10357516; doi:10.3389/fnhum.2023.1121481)
Supplement: Supplementary file 1 [file Table_1.docx]

Supplementary Material

# Supplementary Tables

**Table IV.** Data analysis table for the general systematic review of hand-worn devices. This table breaks down each device by its instrumentation, body part that is targeted, the experiments or aim of the paper that wrote about the device, and the limitations of these devices.

| **Source of Reference** | **Device** | **Instrumentation** | **Targeted Body Part** | **Aim/Experiment(s)** | **Limitations of Device** |
| --- | --- | --- | --- | --- | --- |
| *Adams et al.* (2019) | Commercial Saebo Orthosis with Kinect Sensors | UE kinematic pose in real-time | Arm, wrist, and finger joints | (1) VR-produced indices  (2) Gold-standard tests of UE motor performance | Commercial orthosis is expensive |
| *Avanzino et al.* (2011) | Sensor-engineered glove (Glove Analyzer System) | Spatial and temporal sensors | Individual fingers | (1) Maximum voluntary contraction  (2) Changes in motor performance and cortical excitability during and after a 5 min-finger motor sequence  (3) Recovery dynamics of motor performance and cortical excitability  (4) Control: spinal excitability | Not a one-size fits all glove |
| *Bernocchi et al.* (2018) | GloReha Lite glove | Electrical actuators | Individual finger joints | Hand rehabilitation home-program for two months | (1) Limited application because device cannot be worn by patients with an index of Ashworth spasticity of the hand > 3  (2) Limited time of use because motors will fail after prolonged usage |
| *Bhagubai et al.* (2021) | Experimental sensing system | IMU sensors | Sternum, shoulder, upper/lower arm, hand, thumb, index, and middle fingers. | Fugl-Meyer Assessment of Upper Extremity Tests | Potential error in sensor-to-segment calibration protocol and the global frame definition |
| *Biggar et al.* (2016) | Assistive glove | Elastic bands to aid with finger extension | Hand and fingers | Motion Analysis of fingers both with and without the device for certain tasks: (1) grasping (2) object manipulation | (1) Delayed period of acquisition causes difficulty in using this system as a supportive device in daily activity  (2) Alters natural grasping configuration |
| *Bisio et al.* (2017) | Sensor-engineered glove (Glove Analyzer System) | Spatial and temporal sensors | Individual fingers | Two interventions: (1) Action observation from peripheral nerve stimulation  (2) Motor training protocol | Not a one-size fits all glove |
| *Bonassi et al.* (2020) | Sensor-engineered glove (Glove Analyzer System) | Spatial and temporal sensors | Individual fingers | Experimental finger-opposition training program that lasts two weeks and there are assessments at the beginning and end of the training program. | Not a one-size fits all glove |
| *Bonzano et al.* (2013) | Sensor-engineered glove (Glove Analyzer System) | Spatial and temporal sensors | Individual fingers | Repetitive finger opposition movements of thumb to index, medium, ring, and pinky. | Not a one-size fits all glove |
| *Burns et al.* (2021) | SensoriGlove | Flex sensors and FlexiForce A101 | Individual fingers | Varying degrees of predetermined angles for each finger joint | Due to the manufacturing process, the whole glove would have to be replaced if a sensor was damaged. |
| *Cavallo et al.* (2013) | SensHand V1 | IMU sensors | Forearm, distal phalange of the thumb, index, and middle finger | Analysis of movements of the upper limbs while the participants completed pronation-supination of the forearms, opening/closing of the hands, thumb-forefinger tapping, thumb-middle finger tapping, tremor at rest, and postural tremor. | Surrounding magnetic fields can cause calibration errors. |
| *Chen et al.* (2020) | Sensor & Assistive Device | (1) Sensor Glove: flex sensors  (2) Exoskeleton: linear actuators | Individual fingers | Manipulating objects with and without wearing the robotic hand system. | (1) The movement speed of the exoskeleton hand is fixed and therefore cannot accurately match the sensor glove's speed.  (2) The exoskeleton is not a one-size fits all.  (3) The exoskeleton has only one degree of freedom.  (4) The exoskeleton is too heavy for most patients. |
| *Cheng et al.* (2020) | Brain-computer interface-based soft robotic glove | Air pressure actuators | Individual fingers excluding the thumb | Intervention was over the course of 6 weeks. Each week, the participants participated in standard therapy and experimental therapy (with or without the BCI integration with the glove depending on the group). | They were able to compare effects of using the BCI-assisted motor imagery in robotic rehabilitation but lacked a comparison for decoupling the effects of the robotic rehabilitation. |
| *Coffey et al.* (2014) | Pneumatic exercise glove (PneuGlove) | Inflatable air bladders | Whole hand | Active and passive trials were conducted with the glove on, but depending on the trail, the glove will either be activated or not. | The glove does not assist with individual fingers but effects the whole hand at the same time. |
| *Crema et al.* (2022) | GloReha Glove | Electrical actuators | Individual finger joints | Testing the impact of FES treatment against an assistive device over the course of 9 weeks | (1) Limited application because device cannot be worn by patients with high hand spasticity  (2) Limited time of use because motors will fail after prolonged usage |
| *de Araujo et al.* (2011) | Exoskeleton & static orthosis with a Lycra glove | (1) Passive stabilization of arm and hand  (2) Electromechanical actuator connected to tendons | Arm, wrist, and fingers | Physical therapy rehabilitation program lasting 8 weeks | This device does not interact with the individual finger joints, but the finger. |
| *Dimbwadyo-Terrer et al.* (2016) | CyberTouch | Resistive bend-sensing technology | Individual finger joints | Reaching for objects in a virtual environment | (1) Not a one size fits all glove  (2) expensive |
| *Fei et al.* (2021) | Data glove system | IMU sensors | Individual finger joints | Mimicking tasks from a virtual environment while wearing the glove. | Potential error in sensor-to-segment calibration protocol and the global frame definition |
| *Fischer et al.* (2016) | X-Glove | Linear actuators with tension sensors | Individual finger joints | Intervention protocol over 5 weeks | Device only has two modes: full extension or no extension. |
| *Friedenberg et al.* (2022) | Functional electrical stimulation system | Electrode array | Any targeted body part | Calibration of electrode energization pattern with an associated body part. | Error in the scoring process of the proposals could lead to uncomfortable or painful contractions |
| *Friedman et al.* (2014) | MusicGlove | Flexiforce sensors | Individual fingers | IsoTrainer intervention over 2 weeks | Only measures grip force. |
| *Fu et al.* (2019) | (1) Kinematic sensor glove  (2) Input mitten | (1) Kinematic sensors  (2) Bend sensor | Individual fingers | Videogame therapy for 12 weeks where a sensor glove on unimpaired hand controls FES on the impaired hand to initial a response that is monitored by an input mitten. | Only measures movement, not pressure |
| *Gallo et al.* (2017) | Multimodal haptic device | (1) Liquid Actuators  (2) feedback sensors | Hand, individual fingers, and fingertips | The aim is to provide evidence of a closed-loop system for feedback and assistance in hand function. | Device in bulky and can restrict movement. |
| *Galloway et al.* (2019) | Multi-segment reinforced actuators and applications | Soft, segmented actuators | Individual thumb joints | The aim is to provide evidence of an actuator that will assist all joints of a finger. | The hardware for this system must be strapped to the person's waist, which can be uncomfortable or heavy. |
| *Gentner et al.* (2009) | Würzburg glove | Resistive bend sensors (Flexpoint) | Individual finger joints | Test with and without the glove: (1) modeling a mass into a cylinder and grip it  (2) keeping the hand flat | (1) Not a one size fits all glove  (2) low of sensitivity when bent in the opposite direction |
| *Gerber et al.* (2016) | YouGrabber | Magnetometers and accelerometers | Thumb, index, and middle | 8 different exergames for upper limb training | (1) discomfort or pain from the silicone rings and the neoprene gloves  (2) error in movement tracking when calibration is flawed |
| *Golaszewski et al.* (2012) | Supra-threshold mesh-glove | Electrical stimulation | Whole hand | Stimulation or sham-stimulation was applied for 30 minutes | Can be painful or cause discomfort. |
| *Golomb et al.* (2010) | 5DT sensor glove | Range of Motion from bend sensors | Individual fingers | Videogame therapy focusing on finger flexion/extension. | The glove was not designed for rehabilitation and to be used on spastic hands. |
| *Guo et al.* (2022) | Soft robotic glove | Extendable joint actuators and rigid bone parts | Individual finger joints | Intervention tested the gloves effectiveness by itself and with a BCI protocol. | Binary actuators (e.g., hand open and close) |
| *Hoffman et al.* (2017) | Orthotic device | (1) Support sections (2) electrodes | Forearm, hand, and fingers | The aim is to provide evidence of a glove that not only provides support of the fingers, but also gives electrical stimulation to the hand. | This glove cannot monitor physical responses to the electrical stimulation. |
| *Huynh et al.* (2019) | Custom-made sensor glove | Flex sensors and vibrotactile stimulation motors | Individual fingers | Tested the glove's feasibility to control a custom-made robotic glove and give tactile feedback when grasping an object. | Intrinsic delays disrupted the illusion of the robot hand being the participants' own hand. Therefore, tactile feedback was delayed. |
| *Hwang et al.* (2022) | Self-developed data gloves | IMU sensors | Individual finger joints | Detection of hand tasks (i.e., thumb and grip tasks) | The glove can only be used to evaluate patients with minor motor impairments. |
| *Iwamuro et al.* (2015) | X-Glove | Linear actuators with tension sensors | Individual finger joints | Complete tasks with the paretic hand with and without the glove | The glove's fingertip locations become shifted towards the outer edge of the workspace. |
| *Jarque-Bou et al.* (2020) | CyberGlove Systems | Resistive bend-sensing technology | Individual finger joints | Complete tasks of reaching for objects and manipulations of objects | (1) Not a one size fits all glove  (2) expensive |
| *Jiryaei et al.* (2021) | Soft pneumatic-robotic glove | Air pressure actuators | Individual fingers excluding the thumb | Evaluation of the range of motion in fingers when gripping objects | (1) Thumb is fixed  (2) not delicate manipulation |
| *Jung et al.* (2017) | RAPAEL Smart Glove | Accelerometer and bend sensors | forearm, wrist, and individual fingers | Testing the impact of game-mediated therapy on stroke survivors' recovery | No mean change in the functional assessment tests can imply the negative effects of removing the game-mediated therapy. |
| *Kamockij et al.* (2021) | Virtual reality glove | IMU sensors | Hand and individual fingers | The aim is to develop a system that can motivate the patient and record measurements for clinicians to use for tracking treatment progress. | Calibration of glove may be hindered by external magnetic fields. |
| *Kattenstroth et al.* (2018) | Custom-made stimulation-gloves | 20 Hz electrical stimulation from built-in electrodes | Individual fingertips | Intervention (with/without stimulation) for 3.5 weeks | System was not tested on severe impairment of the hand. |
| *Khallaf et al.* (2017) | Assistive Wrist/finger extension | Gradual extension (Inflatable) | Wrist, metacarpophalangeal, and interphalangeal joints | A task specific, intensive training program that consists of repetitions and structured biofeedback for 16 weeks. | Focus on the whole hand extensions at the same time - not the individual fingers. |
| *Kim et al.* (2022) | (1) 4-DOF soft robotic glove  (2) sensor glove | (1) Passive and active exotendons (2) bend sensors | (1) Thumb, index, and middle (2) Thumb and index | Testing various combinations with the sensor gloves and soft robotic glove. | The sensor glove only monitors the thumb and index, while the assistive device only assists the thumb, index, and middle finger. |
| *King et al.* (2011) | BCI-hand orthosis system | Electrogoniometers | Attached to middle finger | Evaluation of the systems performance by having participants perform repetitive grasping tasks. These tasks completed with the unaffected hand will initiate a grasping response in the orthosis. | The sensor glove only monitors the thumb and index, while the assistive device only assists the thumb, index, and middle finger. |
| *Knutson et al.* (2009) | Custom-built command glove | Bend sensors | Thumb, index, and middle | Testing feasibility of the contralaterally controlled functional electrical stimulation system by performing 55-minute exercise sessions for 12 weeks. | This system did not target all the fingers, but instead the muscles controlling the fingers. |
| *Krukowska et al.* (2014) | Electrode-glove | Electrical stimulation | Extensor muscles of the hand | Testing the effects of the electrode-glove compared to standard bipolar surface electrodes. | Only targets three muscles on the hand. |
| *Lee* (2014) | CyberGlove | Resistive bend-sensing technology | Individual finger joints | The aim is to create a system that will monitor and score hand movement with the use of a sensor glove. | This system requires a commercial glove for use. |
| *Lemos et al.* (2017) | Iglove | IMU Sensors | Hand and fingers | Usability study on the glove's ability to register fine finger movements. | (1) The validation was only for experimental designs where the finger movements are relative to the hand are negligible.  (2) The glove's metrics still need to be validated. |
| *Leuthardt et al.* (2020) | Brain-computer body movement assistance devices | Electrical actuators | Two fingers grouped together (e.g., index and middle, ring and little finger) | The aim is to develop a BCI system that will track rehabilitation progress and control an external assistive glove | The glove does not assist with individual fingers but groups the fingers together. |
| *Li et al.* (2016) | Micro-sensor motion capture system | 9-axis sensor that combines gyroscope, accelerometer, and magnetometer | Shoulder, elbow, torso, and hand | Testing the system’s ability to assess upper-limb movements quantitatively. | This system does not track individual finger movements. |
| *Lieber et al.* (2022) | Pediatric hand exoskeleton (PEXO) | soft three-layered spring blade mechanism for flexion and extension | The four fingers together and the thumb separately | Tested the usability of the glove by having participants complete two functional assessments with and without the glove. | The glove cannot control the index, middle, ring, and little finger separately. |
| *Lin et al.* (2014) | Mesh glove | Electrical stimulation | Whole hand | Intervention that combined mirror therapy with the mesh glove stimulation to see how it benefits the participant in recovering function. | This study only contained individuals with mild to moderate hand impairments. |
| *Linderman et al.* (2012) | Sensor glove | EMG electrodes | Hand muscles | The aim is to develop a sensor glove that can be synced with EEG measurements. | This system lacks finer movements because it only records 3 channels of EMG. |
| *Luhmann et al.* (2022) | Hand exoskeleton | Elongated motion unit | Metacarpophalangeal joints of two fingers | The aim is to develop an exoskeleton that will assist with rehabilitation practices and everyday activities. | This system only assists with two fingers (index and middle), but not the whole hand. |
| *Mawase et al.* (2020) | Ergonomic device | Isometric force | Each fingertip including thumb | This study tested two different finger functions related to force (i.e., maximal voluntary contraction force and individuation) | (1) only measured force - no flexion  (2) only measured the downward force of the finger - not applied force on an object. |
| *Merians et al*. (2009) | (1) CyberGlove  (2) CyberGrasp  (3) Haptic Master | (1) Resistive bend-sensing technology (2) force-reflecting exoskeleton  (3) arm stimulations by force-controlled robot | Arm, hand, individual fingers | Testing the amount of impact, a system that combines monitoring and feedback has on a stroke patients' recovery. | The Haptic Master and the CyberGrasp are bulky and can affect natural movement. |
| *Mohan et al.* (2018) | i-Glove | IMU sensors | Wrist, index, middle, and thumb | Calibration study of the sensors on the glove. | This glove does not monitor all the hands. |
| *Oess et al.* (2010, 2012) | NeuroAssess Glove | Flexpoint sensors | Wrist, metacarpophalangeal, proximal interphalangeal and distal interphalangeal joints of the index finger and thumb | This study tested the reliability of the glove in characterizing the patient's hand function. | (1) The glove is stiff and can restrict movement and handling objects is hard because the glove causes the objects to slip from the hand.  (2) The glove does not monitor the middle, ring, and little finger. |
| *Osuagwu et al.* (2020) | Soft extra muscle glove by Bioservo Technologies AB | (1) tactile sensors (2) artificial tendons with electrical motors | Thumb, middle, and ring finger | This study's focus was to assess whether self-administered home-based hand therapy using the glove can lead to improvement in hand function. | The glove only focuses on three fingers. |
| *Padilla-Magana et al.* (2022) | CyberGlove II | Bend and abduction sensors | Wrist and individual finger joints | Testing feasibility to use the CyberGlove II to assess impaired hand function. | (1) Not a one size fits all glove  (2) expensive |
| *Pan et al.* (2021) | Motion capture system | IMU sensors and surface electromyography | Waist, upper arm, forearm, and hand | Testing feasibility of this system's use to assess impaired upper extremity movement. | No finger monitoring |
| *Patane et al.* (2022) | Hand Test System | Copper and tin conductive sensors | Fingertips | Correlation of the system’s ability to be used to assess impairment in fingers. | (1) Due to the cross-sectional design, the system's pretreatment parameters could not be evaluated.  (2) The variables from the glove cannot be sub-analyzed due to small sample size. |
| *Ranganathan* (2017) | CyberGlove | Resistive bend-sensing technology | Individual fingers | Testing the integration of a sensor glove into a body-machine interface. | (1) Not a one size fits all glove  (2) expensive |
| *Salchow-Hommen et al.* (2019) | Portable IMU-based sensor system | IMU sensors | hand and individual finger joints | Testing feasibility to track finger movements with the glove. | Measurements were affected by the surrounding magnetic fields. |
| *Sallum et al.* (2015) | Orthosis system | Brace component and actuators | One or more fingers of the hand | The aim is to develop a system that can aid in everyday tasks or therapy. | There are no examples of all the fingers being attached to the actuators - only one or two. |
| *Schwarz et al.* (2021) | Motion capture system | IMU sensors | Torso, bicep, wrist, and hand | A cross-sectional observational study testing the validity of the metrics from the system with a clinical assessment. | No finger monitoring |
| *Signori et al.* (2017) | Sensor-engineered glove | Force sensors | Individual fingers | This study aims to define age- and gender-data for finger motor performance. | Glove might not fit all hand sizes. |
| *Sivak et al.* (2009) | P5 Glove | Infrared tracker and piezoelectric bend sensors | Arm, hand, individual fingers | This study tests a VR systems feasibility in hand rehabilitation protocols. | The system was not accurate on virtual movements, which caused slower times of completion of tasks. |
| *Sullivan et al.* (2015) | Glove electrode | Sensory amplitude electrical stimulation | Hand | A pilot study that tests the glove’s ability to be used in home-based intervention. | The glove was difficult to use. |
| *Syeda et al.* (2022) | 5DT sensor glove | Range of Motion from bend sensors | Individual fingers | This study tested the glove’s ability to monitor upper extremity bimanual movements. | The sensor location was variable in finger tapping measurements. |
| *Thielbar et al.* (2014) | (1) CyberGlove  (2) PneuGlove | (1) Resistive bend-sensing technology (2) Air pressure actuators | Individual fingers | This study tested the impact of finger individuation training with virtual reality. | CyberGlove: (1) Not a one size fits all glove  (2) expensive |
| *Thielbar et al.* (2017) | Electromyography-driven actuated glove | Cable actuation | Individual fingers | This study tested the glove’s ability to be used in stroke interventions. | This system hindered natural movement by its bulkiness and weight. |
| *Triandafilou et al.* (2011) | X-Glove | Linear actuators with tension sensors | Individual finger joints | This study investigated the impact of prolonged and repetitive passive range of motion in individuals effected by a stroke. | Device only has two modes: full extension or no extension. |
| *Uswatte et al.* (2006) | (1) Sling/hand-splint  (2) Half-glove | (1) fixed hand with splint  (2) fingerless gloves | Hand and individual finger | This study investigates the effects of different types of training and restraint used in those training. | These devices are only for stabilizing the hand. |
| *Wachter et al.* (2018) | Sensor Glove | Pressure sensors (FSR 151) | Palm and fingertips excluding the thumb | This study focused on finding the means of a median nerve block stimulation to improve different types of grip forces. | The thumb is being excluded from applied-pressure monitoring. |
| *Wijesundara et al.* (2021) | Assistive glove | Hydraulic actuators | Individual fingers | The aim is to develop a hydraulic actuator that mimic the articulation of the human hand. | This assistive device is a glove and therefore, sliding the glove onto a spastic hand will be difficult especially with the actuators. |
| *Wille et al.* (2009) | Custom-designed gloves | Bend sensors, accelerometers and magnetometers | Forearm and individual fingers | This study aims to develop a virtual reality based, pediatric interactive therapy system for children with neuromotor disorders. | Therapist is needed because the system is designed for simplicity and not accuracy. |
| *Wolbrecht et al.* (2018) | FINGER robotic exoskeleton device | Load cells and the robot responses to the load cells inputs | Index and middle finger | This study aims to find the contributions of finger weakness and reduced individuation of fingers in stroke patients. | (1) Cannot be used with severe impairment  (2) Does not utilize the thumb or other fingers (i.e., ring and little finger) |
| *Yap et al.* (2016) | MR-Glove | Soft pneumatic actuators | Individual fingers | This study focused on the evaluation of the gloves use with functional magnetic resonance imaging in hand rehabilitation tasks. | This glove cannot be used to assist in unclenching the hand or extending the fingers of individuals with severe spasticity. |
| *Yeow et al.* (2019) | Assistive glove | Hydraulic actuators | Individual fingers | The aim is to develop hydraulic actuators comprised of bending actuators to be used in multiple applications. | This assistive device does not allow for graded flexion, extension, and grip tasks. |
| *Yurkewich et al.* (2020) | Myoelectric untethered robotic glove | actuator and cable tie tendons | Individual fingers | This study aims to use an assistive robot to help with bimanual activities in stroke patients. | Has to be slipped onto the hand - not an exoskeleton. |
| *Zbytniewska et al.* (2021) | ETH MIKE | End-effector robot | Index finger | This study aims to validate an assistive hand robot system and test its capability in capturing the sensorimotor impairments of the fingers. | (1) Only one finger can be used  (2) One degree of freedom movement assistance |

**Table V.** Data Analysis Table for BCI-based hand-worn devices found in the BCI specific review search. This table breaks down each device by description of hand-worn device, type of Brain Activity Monitoring (BAM) device, purpose of the study, the results from the study, and the limitations of the hand-worn devices.

| **Source of Reference** | **Hand-Worn Device** | **BAM Device** | **Purpose of Study** | **Results** | **Limitations of hand-worn device** |
| --- | --- | --- | --- | --- | --- |
| *Ang et al.*  (2014) | Haptic Knob (HK) Robot; finger extension and flexion; 2 DOF | EEG (27 Channels) | Investigated benefits of using the EEG-based MI-BCI coupled with a haptic knob robot in therapy compared to physical therapy practice of the stroke-impaired extremity. | Significantly greater FMMA scores from the BCI-HK group compared to the control group. | Motor improvements measured in FMMA are limited the device focusing more on the proximal arm. Therefore, gains can not be directly related to ADLs. |
| *Barsotti et al.* (2015) | BRAVO Exoskeleton; 5 planar mechanisms driven by 2 motors (1 for thumb & 1 for all the other fingers) | EEG (13 Channels) | Testing the feasibility of using this system on stroke patients to allow them to perform self-initiated and controlled grasping movements. | System was successful in initiating robotic assistance using patients volitional effort. | This study did not evaluate the performance metrics of the system during the exercise. |
| *Bauer et al.*  (2015) | Amadeo Hand Robot; 5 mechanical sliders to extend each finger | EEG (31 Channels) | Aim to determine the connection between MI and ME and how predictable they are. | Brain-robot-interface can activate both MI and ME. | The hand is strapped to the system and the fingers can only move along the sliders, no other DOFs. |
| *Buch et al.*  (2008) | Mechanical orthosis that controls grasping motions for fingers, excluding the thumb | MEG (135-275 Channels) | Testing the systems ability to be used to move completely paralyzed hands. | The system can be used to perform hand grasping motions with a paralyzed hand. | The system did not include the thumb. Therefore, there will be no assisted movement of the thumb. |
| *Bundy et al.*  (2017) | 3-finger pinch exoskeleton | EEG (8 Channels) | Testing the feasibility of using this system to control the stroke patients’ affected hand with the unaffected hemisphere of the brain. | Significant improvement in motor function for the affected hand with this system. | The system did not assistive with movement of the ring and little finger. Their assistive device will only allow for 3 finger-pinch motion. |
| *Cantillo-Negrete et al.* (2015) | Mechanical orthosis that controls finger movement. | EEG (11 Channels) | Testing the systems ability to be controlled by MI signals. | The orthosis is capable of having a good performance as part of a BCI system. | The orthosis can not sense the physical position of the fingers, therefore sensors can be added to give the orthosis the capability to determining the fingers exact position in space. |
| *Caria et al.*  (2020) | Robotic Hand Orthosis; Mechanical actuators for each finger and optical sensors for finger positions | EEG (16 Channels) | Investigating how neural plasticity in the motor networks change in severely impaired chronic stroke patients after BCI-assistive intervention. | Found that BCI-assistive intervention can reinforce ipsi-lesional brain activity and elicit intra- and inter-hemispheric reorganization with proprioceptive function of the affected hand. | The hand cannot fully form a fist. Therefore, it lacks the capabilities of assisting all finger motions. |
| *Carino-Escobar et al.* (2019) | Hand Orthosis; Finger flexion and extension | EEG (11 Channels) | Attempts to describe the cortical activations during BCI interventions and the relationships between ERD/ERS. | The longitundinal analysis of EEG brain rhythms during hand rehabilitation can be valuable for clinical prognosis and BCI intervention goals. | The orthosis excludes the thumb and therefore, it can not assist with typical grasping motions. |
| *Cheng et al.*  (2020) | Soft Robotic Module; 4 pneumatically driven actuators on the fingers with a thumb splint | EEG (24 Channels) | Testing the BCI-Assistive system intervention against standard intervention methods. | The BCI-Assistive system was proven useful in improving motor function compared to the standard methods. | This system on controls the four fingers and does not control thumb movement. The thumb is held stationary and therefore, the wearer has limited grasping movement. |
| *Chowdhury et al.* (2020) | Exoskeleton; mechanical actuators on thumb, index, middle with FSRs on the fingertips | EEG (12 Channels) | Testing the systems ability to be used for activating hand movement before ME based on MI. | Proved that incorporating active hand movement with physical practice and then assisting with movement with MI can improve motor-recovery. | This system does not assist with ring and little finger for movement, therefore the whole hand is not being rehabilitated. |
| *Coffey et al.* (2014) | PneuGlove; whole hand pneumatic actuation | EEG (27 Channels) | Providing information about their BCI-Assistive system and specifics on their devices. | They positively advocate for replication of their system in the hope that researchers will experiment with this type of system to prove that it is benefical for functional recovery. | This system actuates the whole hand and not the individual fingers, therefore only 1 DOF can be obtained. |
| *Frolov et al.*  (2017) | Exoskeleton; pneumatic actuators on all fingers | EEG (30 Channels) | Testing the benefits of using a BCI-controlled exoskeleton and a passive exoskeleton intervention. | Their BCI-controlled exoskeleton proved to be more beneficial with rehabilitation intervention then just passive use of the exoskeleton. | The BCI-Assistive system was used for opening and closing the hand. Therefore, they did not demonstrate individual finger movement with the exoskeleton. |
| *Holmes et al.*  (2012) | IpsiHand Bravo; ExoFlex and linear actuators for all fingers excluding the thumb | EEG (14 Channels) | Presents the IpsiHand Bravo BCI System for long-term rehabilitation. | Their system was descibed to be low-cost and can achieve greater than 90% accuracy in most cases. | This system on controls the four fingers and does not control thumb movement. The thumb is held stationary and therefore, the wearer has limited grasping movement. |
| *Kasashima-Shindo et al.*  (2015) | Motor-driven orthosis with a servomotor to achieve finger extension-flexion movement at 1 joint. | EEG (1 Channel [C3 or C4]) | Test the application of anodal tDCS in BCI training in stroke patients. | They found that Anodal tDCS could be used as a conditioning tool to increase ERD for BCI triggering. | The motor-driven orthosis assists finger movement from all four fingers at once and does not include the thumb. |
| *King et al.*  (2011) | Tendon-driven orthosis glove; index, middle and thumb actuation and electro goniometer on the back of the hand | EEG (63 Channels) | Testing the system on healthy individuals to prove the systems capabilities in contralateral control hand-grasping paradigm. | They found that the EEG-based BCI system with a hand orthosis is feasible. | This system does not assist with ring and little finger for movement, therefore the whole hand is not being rehabilitated. |
| *Li et al.* (2019) | Hand Exoskeleton; mechanical actuator for each finger and force sensors on the tips of the fingers | EEG (3 Channels) | Presents a multi-segment assistive exoskeleton for hand rehabilitation. | This device had an activation rate above 90%. | They only prototyped the glove to be worn on the left hand and was programed to move the fingers at the same time to open/close the hand. |
| *Naros et al.*  (2016) | Amadeo Hand Robot; 5 mechanical sliders to extend each finger | EEG (3 Channels) | Prove that reinforcement learning is best achieved by incorporating BCI tasks with a participants learning experience. | Their system was proven useful in BCI-based therapeutic interventions for hand rehabilitation. | The hand is strapped to the system and the fingers can only move along the sliders, no other DOFs. |
| *Nishimoto et al.* (2018) | Motor-driven orthosis; targeted the metacarpophalangeal and proximal interphalangeal joints and stimulated the forearm for finger extension. | EEG (1 Channel [C3 or C4]) | Prove that their new compact BCI system was feasible as a tool in hand neurorehabilitation. | Their system can be used in clinical settings and BCI training interventions that target brain plasticity and impaired hand function. | The finger extension occurs for all fingers simultaneously. There is no way to extend the fingers individually. |
| *Norman et al.* (2018) | FINGER Robotic Exoskeleton; extends one finger while inhibiting movement in the other) | EEG (16 Channels) | Demonstrates the use of a BCI-controlled finger-individuated robotic hand orthosis will allow for more complex movement training. | Their system showed promise as a tool for enhancing motor function recovery and motor learning for hand tasks. | The FINGER device only attaches to two fingers (index and middle) at a time. Therefore, the device can not assist with all finger movements at once. Additionally, it does not allow for simultaneous movement of the fingers. |
| *Ono et al.* (2016) | Power Assist Hand; pneumatic device for extension and flexion of fingers | EEG (9 Channels) | Studied the transition rate of the successful operation of ERD-BCI and compared the ERD strength when received synchronous and delayed multimodal feedback. | When using the BCI system and synchronous feedback, the ERD strength was the strongest, which means functional recovery was the best. | The hand-worn device is programed to open and close the hand with synchronous movement of the fingers. |
| *Ono et al.* (2018) | Power Assist Hand; pneumatic device for extension and flexion of fingers | EEG (1 Channel [C3 or C4]) | Investigated the benefits of neurofeedback training with realistic proprioceptive feedback with MI-related ERD power. | This system shown potential in being more effective for individuals who need BCI-based rehabilitation for neuroprosthetics | The hand-worn device is programed to open and close the hand with synchronous movement of the fingers. |
| *Ramos-Murguialday and Birbaumer* (2015) | Robotic Hand Orthosis; Mechanical actuators for each finger and optical sensors for finger positions | EEG (128 Channels) | Testing the hypothesis that each motor task (e.g., open/closing hand) will present individual EEG signatures and the EEG activity during the orthosis movement will mimic neural activity during active movement. | They were able to determine EEG features that correspond to motor tasks that are relevant in rehabilitation. | The hand cannot fully form a fist. Therefore, it lacks the capabilities of assisting all finger motions. |
| *Ramos-Murguialday et al.* (2019) | Robotic Hand Orthosis; Mechanical actuators for each finger and optical sensors for finger positions | fMRI | Testing the hypothesis that 4-weeks of BCI-based intervention will promote significant immediate and long-lasting motor function gains. | They proved that BCI-based rehabilitation in chronic stroke patients with severe paresis is very beneficial for motor function recovery and can have long-lasting improvements. | The hand cannot fully form a fist. Therefore, it lacks the capabilities of assisting all finger motions. |
| *Randazzo et al.* (2018) | Mano; hand exoskeleton with artificial tendons that actuate finger movements | EEG (16 Channels) | Aims for their device to overcome limitations with current device on the market and test its ability to be controlled by EEG. | Their device provides realistic feedback to the wearer and is promising for triggering neuroplasticity, which will help in motor function rehabilitation. | Majority of the fingers can be actuated independently, but the ring and little fingers are coupled in their actuation. |
| *Stan et al.* (2015) | Orthosis system; actuates fingers on a linear plane (vertical [4 fingers] or horizontal [thumb]) | EEG (8 Channels) | Tested the effectiveness of controlling the hand with the BCI-Assistive system. | Successfully controlled the orthosis in performing movement tasks. | Since the actuators can only move in a planar motion, the fingers’ movement will be restricted and not as natural. |
| *Tacchino et al.* (2017) | GloReha Glove; electrical actuators on all 5 fingers | EEG (19 Channels) | Investigates the neural correlations between subjects' active participation to functional robotic-based movements through EEG. | Found that coupling early planning for movement and proprioceptive feedback will trigger prolonged brain activity. | (1) Limited application because device can not be wore by patients with an index of Ashworth spasticity of the hand > 3 (2) Limited time of use because motors will fail after prolonged usage |
| *Tsuchimoto et al.* (2019) | Motor-driven robotic orthosis; mechanical actuators to form a pinching motion | Bipolar EEG | Testing the effectiveness of EEG-based neurofeedback intervention against other treatments. | They were able to verify that BCI-Assisted therapy can be extremely useful in improving the motor rehabilitation. | The hand-worn device only assists with two finger pinching motion. |
| *Vukelic and Gharabaghi* (2015) | Amadeo Hand Robot; 5 mechanical sliders to extend each finger | EEG (128 Channels) | Investigating the difference in the beta-activity modulation from either visual feedback or proprioceptive feedback with BCI-based rehabilitation. | Found that proprioceptive feedback resulted in lower variability and consistently maintain and reproduce beta-activity compared to visual feedback. | The hand is strapped to the system and the fingers can only move along the sliders, no other DOFs. |
| *Wada et al.* (2019) | Power Assist Hand; pneumatic device for extension and flexion of fingers | EEG (1 Channel [C3 or C4]) | Testing the effect of having 2 weeks of BCI-assistive rehabilitation intervention compared to the traditional passive therapy. | The BCI-Assistive system was proven superior then traditional therapy because of the use of MI activation in cortical activity. | The hand-worn device is programed to open and close the hand with synchronous movement of the fingers. |
| *Wang et al.* (2018) | Hand Robot; mechanical actuators with 2 DOF | EEG (16 Channels) & fMRI | Testing the hypothesis that BCI-based therapy has the capability of improving motor functional recovery and neuroplasticity. | Found that sustainable motor functional improvement can occur when the patient undergoes proper guided rehabilitation with neurofeedback. Additionally, neuroplasticity can occur in multiple brain networks involving both hemispheres. | The hand robot has the potential of being too heavy for the wearer which can effect the usability in individuals who have muscle fatigue or weakness. |
| *Witkowski et al.* (2014) | Hand Exoskeleton (BioRobotics Institute; Scuola Superiore Sant'Anna, Pisa, Italy); assists with index and thumb movement | EEG (5 Channels) + EOG | Investigating if integrating EOG into BCI-assistive system can improve the control and safety of the hand exoskeleton. | Found that it is possible to use a hybrid EEG/EOG BCI controlled exoskeleton and they can be used to improve the effectiveness of training protocols. | This hand-worn device is limited in its ability to control all fingers since the exoskeleton only targets the thumb and index. |
| *Zhang et al.* (2019) | Soft Rehabilitation Gloves; 5 pneumatic actuators | EEG+EOG (40 Channels) | Introduces a 3-mode interface that controls a soft hand robot to perform repetitive tasks. | This system provides 3 different ways to control the soft glove in real-time. | The soft rehabilitation glove's effectiveness in assisting grasping objects will decrease over time when the pressure within the actuators/robot drops below the barometric threshold. |
